# Structural basis of diadinoxanthin-Chl a/b–binding proteins in the photosystem I supercomplex of *Euglena gracilis*

**DOI:** 10.1126/sciadv.aea5561

**Published:** 2026-03-06

**Authors:** Tianyu Bai, Zhiyuan Mao, Dapeng Sun, Yang Tang, Ziqiu Lu, Fei Ma, Bingxin Ma, Jinghua Chen, Weiwei Wang, Yuelin Li, Shufang Liu, Yi Yang, Yumei Wang, Lirong Tian

**Affiliations:** ^1^ Key Laboratory of Molecular and Cellular Biology of the Ministry of Education, Hebei Research Center of the Basic Discipline of Cell Biology, Hebei Collaboration Innovation Center for Cell Signaling, College of Life Sciences, Hebei Normal University, Shijiazhuang 050024, Hebei, China.; ^2^ Institute of Physics, Chinese Academy of Sciences, Beijing 100190, China.; ^3^ State Key Laboratory of Forage Breeding-by-Design and Utilization, Key Laboratory of Photobiology, Institute of Botany, Chinese Academy of Sciences, Beijing, China.; ^4^ College of Life Sciences, Zhejiang University, Hangzhou 310058, Zhejiang, China.

## Abstract

*Euglena gracilis* is a phototrophic flagellate that has evolved through secondary endosymbiotic events and belongs to green-lineage organisms. We report a unique structure of photosystem I-light harvesting complex I (PSI-LHCI) supercomplex from *E. gracilis* at a 2.23-angstrom resolution by cryo–electron microscopy. The supercomplex is composed of 8 core subunits and 16 LHCIs and exhibits distinctive structural features compared to its counterparts in green algae and plants. The LHCI subunits encircle the core complex, forming a two-layered arrangement that comprises six pairs of tightly packed heterodimers. Specifically, the 16 LHCIs consist of 4 diadinoxanthin-chlorophyll (Chl) a/b–binding antennae (Lhcbm) and 12 diadinoxanthin-Chl a–binding antennae (Lhca), and they exhibit characteristic pigment compositions combining features of green- and red-lineage organisms. These findings provide a robust structural foundation for elucidating the mechanisms of light harvesting and energy transfer as well as insights into the evolutionary changes of green-lineage PSI-LHCI.

## Teaser

The high-resolution *Euglena* PSI structure reveals how diverse two-layered antennas adapt to secondary endosymbiotic evolution.

## INTRODUCTION

Oxygenic photosynthesis in land plants, algae, and cyanobacteria is driven by two large pigment-protein complexes, photosystems I and II (PSI and PSII, respectively). The PSII complex catalyzes water oxidation to produce oxygen, while PSI drives the transfer of electrons derived from water oxidation to ferredoxin, leading to the generation of reducing power necessary for the reduction of CO_2_ into sugars. In eukaryotic photosynthetic organisms, the PSI core is surrounded by light-harvesting complex I (LHCI), forming a PSI-LHCI supercomplex. The core subunits of PSI are relatively conserved during evolution, although some have been lost or emerged because of differences in the peripheral antennae they bind, whereas the quantity, arrangement, protein composition, and bound pigments of LHCI exhibit substantial variability across different photosynthetic organisms, which likely reflects the adaptive evolution in response to distinct light environments experienced by different species.

The primary oxygenic eukaryotes, including red algae, green algae, and glaucophytes, are formed through the endosymbiotic uptake of a cyanobacterial cell into a eukaryotic host cell. Subsequently, secondary endosymbiotic events involving the engulfment of the most primitive red and green algae led to the emergence of the “red” and “green” clades (*1*, *2*). A branch of the “green” clade gives rise to the green algae, moss, and land plants. Meanwhile, green algal secondary endosymbiosis gives rise to Euglenophytes, Chlorarachnophytes, and “green” dinoflagellates (*3*). Now, the structures of PSI in red algae (*4*–*6*), green algae (*7*–*12*), moss (*13*–*15*), higher plants (*9*, *16*–*21*), glaucophytes (*22*), and red-lineage algae originating from secondary endosymbiosis including diatoms (*23*), dinoflagellates (*24*, *25*), and haptophytes (*26*) have been elucidated. However, the structure of PSI within the green lineage that has undergone secondary endosymbiosis remains unknown.

*Euglena gracilis* is a freshwater photosynthetic flagellate belonging to the phylum Euglenophyta (*27*). It originates from a secondary endosymbiotic event and is part of the green lineage. Unlike the chloroplasts of higher plants and most green algae that are enveloped by two membranes, the chloroplast of *Euglena* has three membranes (*28*, *29*). In particular, the LHCI and LHCII (previously known as LHCPII or pLHCP II) of *E. gracilis* are translated from large mRNAs into polyprotein precursors. These precursors consist of multiple, concatenated LHC subunits that are subsequently posttranslationally targeted to the chloroplast and cleaved into individual LHC proteins (*30*–*33*). Recent molecular phylogenetic studies suggest that *E. gracilis* may have also acquired some red-lineage genes through lateral gene transfer from algal sources other than the green algal endosymbiont (*34*). Consequently, its nuclear genome contains genes originating from either green or red algae (*34*). *Euglena* has a chlorophyll (Chl) a/b antenna system similar to that of green algae and plants. Its LHCI (Lhca, *Lhca* gene products) and LHCII (Lhcb, *Lhcb* gene products) polypeptides are closely related to those of green algae (*35*, *36*). Notably, *Euglena* contains unique carotenoids (Cars): diadinoxanthin (Ddx) and diatoxanthin (*36*–*38*), which are found exclusively in red-lineage algae, such as diatoms (*23*), dinoflagellates (*24*, *25*), and haptophytes (*26*). However, it lacks lutein (Lut) and violaxanthin, which are found in higher plants and green algae (*7*, *8*, *19*). Therefore, the classification and evolutionary position of *E. gracilis* remain a subject of debate.

Although *Euglena* occupies such a crucial position on the evolutionary branches, the photosynthetic apparatus of *Euglena* has not been characterized thus far. Here, we solved the structure of PSI-LHCE from *E. gracilis* using single-particle cryo–electron microscopy (cryo-EM) at a resolution of 2.23 Å. Hereafter, we adopt the recently proposed nomenclature, LHCE (“E” stands for *Euglena*), for these lineage-specific antenna proteins (*39*). Our findings provide crucial insights into the unique structural arrangement and energy transfer features of the PSI-LHCE supercomplex in *E. gracilis*, which not only provide important clues into the organization and energy transfer mechanism of LHCE in green secondary endosymbiotic algae but also offer a basis for elucidating the origin and evolutionary status of *Euglena*.

## RESULTS

### Overall structure of PSI-LHCE

**Fig. 1.**
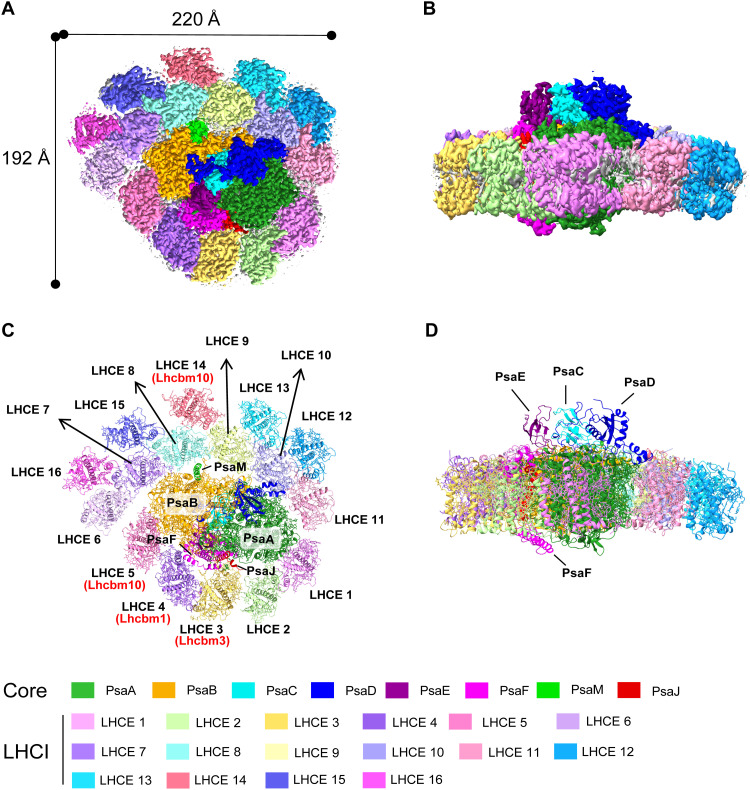
Overall structure of the *Euglena* PSI-LHCE supercomplex. (**A** and **B**) Cryo-EM density map of the PSI-LHCE supercomplex viewed from the stromal side (A) and its side view (B). (**C** and **D**) Structure of the PSI-LHCE supercomplex viewed from the stromal side (C) and its side view (D).

The PSI-LHCE supercomplex from *E. gracilis* was purified by sucrose density gradient ultracentrifugation and size exclusion chromatography (fig. S1, A and B). The peptide composition, spectroscopic properties, and pigment compositions of them are shown in fig. S1 (C to F). These analyses confirmed that the purified samples were sufficiently pure and contained Chl a, Chl b, Ddx, and β-Car as main pigments. Using single-particle cryo-EM, we solved the PSI-LHCE supercomplex structure at a global resolution of 2.23 Å on the basis of the “gold standard” of Fourier shell correlation of 0.143 (fig. S2 and table S1). The overall structure of *E. gracilis* PSI-LHCE comprises a PSI core complex and a peripheral antenna system containing 16 Ddx-Chl a/b–binding proteins arranged in a ring surrounding the core. This arrangement markedly differs from the structures reported for plants and green algae (Fig. 1). The whole PSI-LHCE supercomplex resembles the shape of a bear’s paw when viewed from the stromal side (Fig. 1A). The PSI core comprises only eight subunits (PsaA/B/C/D/E/F/J/M), whereas the 16 LHCE antenna subunits surrounding the PSI core are organized into two distinct belts (Fig. 1C). The first LHCE belt is positioned at the PsaB/F/J/A side and consists of five LHCEs (LHCEs 1 to 5). The second LHCE belt, located opposite to the PsaB/F/J/A region, contains 11 LHCE subunits (LHCEs 6 to 16) arranged in a semicircular, two-layer configuration. The inner layer comprises six LHCEs (LHCEs 6 to 11), whereas the outer layer consists of five LHCE subunits (LHCEs 12 to 16). The high-quality cryo-EM map and subsequent sequence analysis during model building identified 12 Lhca proteins (LHCEs 1, 2, 6 to 13, 15, and 16) and 4 Lhcbm proteins (LHCEs 3 to 5 and 14) in the PSI-LHCE supercomplex (Fig. 1C).

In addition to the protein subunits, we identified 276 Chls a, 6 Chls b, 13 β-Car, 41 Ddx, 3 Fe_4_S_4_ clusters, 4 phylloquinones (PQNs), and 24 lipids on the basis of the clear density map obtained (fig. S3 and table S2). Collectively, this gives rise to a supercomplex containing 24 protein subunits and 367 cofactors, with an overall molecular weight of 770 kDa. In addition, four incomplete and discrete density regions are identified within the membrane bilayer (fig. S4A).

### PSI core complex

**Fig. 2.**
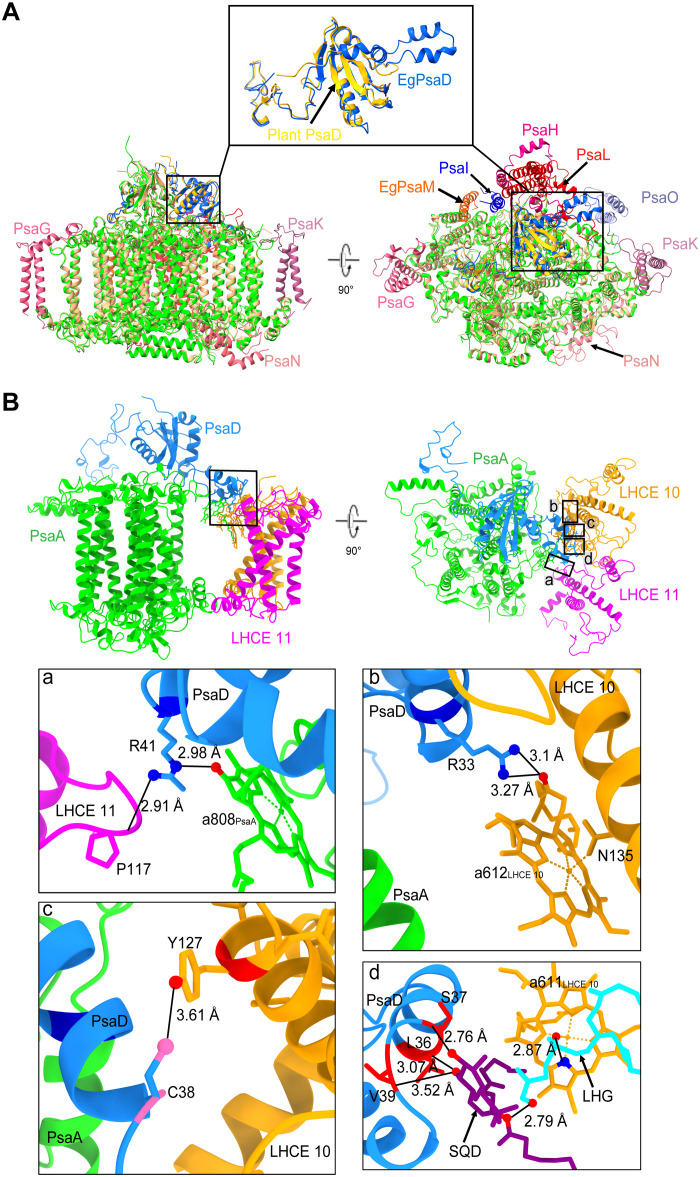
Comparison of PSI core structures between *E. gracilis* and plants. (**A**) Comparison of the PSI core structure between *E. gracilis* and maize (PDB code: 5ZJI), with the structure of PsaD enlarged in the upper part. The conserved PSI cores of plant and *E. gracilis* are colored in wheat and green, respectively, and the special subunits of plant PSI core (PsaG/H/I/K/N/O) and PsaD/M of *E. gracilis* are colored differently. (**B**) Interactions between PsaD and PsaA, LHCE 10, and LHCE 11 subunits in PSI-LHCE. Enlarged views of the interactions among these subunits are presented in panels a to d. Color: LHCE 10, LHCE 11, PsaD, and PsaA are colored orange, magenta, dodger blue, and lime, respectively. Atoms: N, blue; O, red; S, hot pink. Cofactors: SQD, purple; LHG, cyan.

The *E. gracilis* PSI core contains only five transmembrane subunits (PsaA/B/F/J/M) and three extrinsic subunits (PsaC/D/E) located on the stromal side, forming a highly compact PSI core. Similar to the structure of PSI reported in *Dunaliella salina* (*10*), *E. gracilis* PSI retains the seven most central protein subunits, in addition to an extra PsaM that is absent in *D. salina* (fig. S5). The PsaG/H/I/K/L/O/N subunits located at the outermost edge of the green-lineage PSI core have all been lost in *E. gracilis* (Fig. 2, fig. S5, and table S2 and S3). The PsaG/H/J/K/L subunits are not found in the genomes of *E. gracilis* or in other available *Euglena* genomes, whereas PsaI exists in some *Euglena* species (e.g., *Euglena viridis*, *Euglena clara*, and *Euglena mutabilis*), but it was not identified in the genome of *E. gracilis*.

The structure of most core subunits in *E. gracilis* is highly conserved with its counterpart in the green-lineage photosynthetic organisms (fig. S5). However, *E. gracilis* PsaD exhibits specific structural properties compared to its homologous counterpart in both green- and red-lineage organisms (figs. S5 and S6). *E. gracilis* PsaD contains two small α helices near its N terminus (Fig. 2A), and they are located close to the thylakoid membrane at a junction between LHCE 10, LHCE 11, and PsaA (Fig. 2B), which may play a crucial role in facilitating the interactions between LHCE 10, LHCE 11, and the PSI core. At the interface among LHCE 10, LHCE 11, PsaD, and PsaA, two lipid molecules—SQDG (sulfoquinovosyl diacylglycerol, labeled as SQD in the figure) and PG (1,2-dipalmitoyl-phosphatidyl-glycerole, labeled as LHG in the figure)—are found and may mediate the connection between LHCE 10 and PsaA via amino acid residues on the two small α helices of PsaD. The N-terminal amino acids Val^39^ (V39), Leu^36^ (L36), and Ser^37^ (S37) of PsaD interact with SQDG via hydrogen bonds (Fig. 2B-d). SQDG, in turn, links PsaD to PG via hydrogen bonding (Fig. 2B-d), and PG further interacts with the Chl a611 of LHCE 10 (Fig. 2B-d). In addition, PsaD directly interacts with LHCE 11 via a hydrogen bond between Arg^41^ (R41) and Pro^117^ (P117) and with LHCE 10 between Cys^38^ (C38) and Tyr^127^ (Y127) (Fig. 2B-a and c). Moreover, LHCE 10/Chl a612 forms hydrogen bonds with Arg^33^ (R33) of PsaD and Asn^135^ (N135) of LHCE 10 (Fig. 2B-b), thereby enhancing the stability of the complex.

**Fig. 3.**
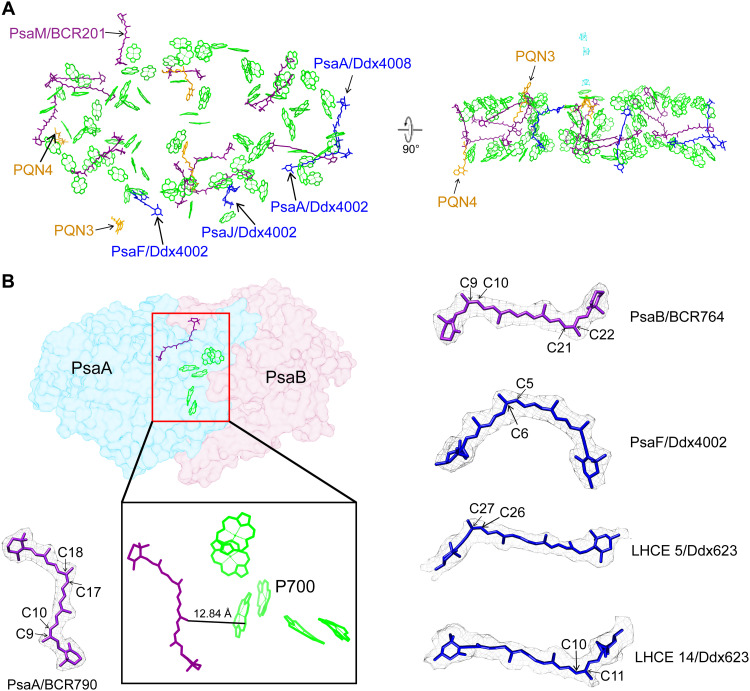
Special cofactors and *cis*-Cars in *E. gracilis* PSI-LHCE. (**A**) Arrangement of major cofactors in the PSI core. All Chls a and β-Cars in the PSI core are colored green and purple, respectively, whereas four Ddx and four PQNs are colored blue and orange, respectively. (**B**) *cis*-Cars in the PSI-LHCE supercomplex. Within the high-resolution structure of the PSI-LHCE supercomplex, a total of five *cis*-Car molecules are identified: two *cis*-β-carotene and three *cis*-Ddx molecules. The *cis*-carotene at the PsaA/BCR790 binding site is located close to the P700 reaction center.

The PSI core complex comprises 88 Chl a, 13 β-Car, and 4 Ddx molecules (Fig. 3A). Most of the pigment-binding sites are conserved in the green lineage (fig. S7A). Particularly when compared to the minimal PSI core of *D. salina* (*10*), nearly all pigment-binding sites remain conserved (fig. S7A). However, four Ddx (designated as PsaF/Ddx 4002, PsaJ/Ddx 4002, PsaA /Ddx 4002, and PsaA/Ddx 4008, in accordance with the *D. salina* PSI-LHCI structure) were identified in the *E. gracilis* PSI core on the basis of the high-quality cryo-EM map (Fig. 3A, fig. S7B, and table S2). These Ddx molecules substitute for β-Car in the PSI core of *D. salina* (*10*) and other green-lineage organisms (fig. S7, B, D, and F) (*7*, *8*, *13*, *19*). Notably, the positions corresponding to PsaJ 4002 and PsaA 4008 are also occupied by Ddx in diatoms (fig. S7G) (*23*) but not in dinoflagellate (fig. S7H) (*24*, *25*). These Ddx molecules are located at the interface between the peripheral antennae and the core subunits, a distinct position suggesting their potential involvement in light harvesting or photoprotection of the PSI core (fig. S7I). Moreover, a special β-Car (PsaM/BCR201) is bound near PsaM, positioned between LHCE 7 and the PsaB subunit, and extends across the entire thylakoid membrane from its head to tail (Fig. 3A and fig. S7I). This pigment has not been identified in plants (*9*, *19*–*21*), green algae (*7*, *8*), red algae (*4*–*6*), cryptophytes (*40*), and other related species (fig. S7, B, D, and F) (*13*, *15*) but is present at the same location in diatoms (*23*), dinoflagellates (*24*, *25*), and haptophytes (fig. S7, G and H) (*26*). The conservation of Ddx and PsaM/BCR201 binding in the core complex between *E. gracilis* and diatoms, dinoflagellates, and haptophytes suggests that these special Car-binding sites may be a result of secondary plastid endosymbiosis.

Furthermore, on the basis of high-quality electron density, we resolved five *cis*-Cars in the PSI-LHCE supercomplex: two *cis*-β-Car (9,17-*cis* β-Car_PsaA/BCR790_ and 9,21-*cis* β-Car_PsaB/BCR764_) and one *cis*-Ddx (5-*cis* Ddx_PsaF/Ddx4002_) in the core, as well as two *cis*-Ddx (26-*cis* Ddx_LHCE 5/Ddx623_ and 10-*cis* Ddx_LHCE 14/Ddx623_) in the LHCE antennas (Fig. 3B). These *cis*-Cars may play an important photoprotective role. Notably, the *cis*-β-Car at PsaA/BCR790 is located exceptionally close to the P700 reaction center at a distance of 12.84 Å, which may play a crucial photoprotective role by quenching the reaction center’s triplet state (*16*, *41*–*43*).

We also identified two additional PQNs (designated as PQN3 and PQN4) in the PSI core. PQN3 is located between LHCE 4 and the PSI core, and PQN4 is positioned between LHCE 5 and the core (Fig. 3A and fig. S4B). The head group of PQN3 faces the stromal side, whereas the head group of PQN4 orients toward the lumenal side. To date, these two PQNs have not been observed in the PSI core of any other green- or red-lineage organisms. In the reaction center, PQNs typically act as secondary electron acceptors (A_1_), receiving electrons from A_0_ and transferring them to the Fe_4_S_4_ cluster. However, given the relatively long distance of PQN3 and PQN4 from the electron transfer chain, it is unlikely that they participate directly in the electron transfer process of the reaction center. The presence of these noncanonical PQNs is reminiscent of the discovery of the auxiliary plastoquinone Q_C_ site in PSII (*44*), as both Q_C_ and PQN3/PQN4 represent binding sites for quinone molecules outside the established linear electron transport chain. However, unlike Q_C_, which was proposed to reside near the mobile Q_B_ exchange channel on the cytoplasmic side, PQN3 and PQN4 are structurally embedded between the LHCE and the core. Furthermore, unlike the two PQNs in the reaction center, PQN3 and PQN4 are not deeply buried within the thylakoid membrane but are positioned closer to the stromal side and lumenal side, respectively. Given that PQN3 and PQN4 are located in the larger gaps between the peripheral antenna and the core, they do not show distinct interactions with surrounding amino acids. The density around these PQNs also suggests a large quantity of unidentifiable lipid molecules. Therefore, these two additional PQNs may play a structural stabilization role in anchoring the LHCE to the PSI core, a feature that may be a unique product of the *Euglena* plastid’s secondary endosymbiosis.

### Location and structures of LHCE subunits

The 16 LHCE subunits surrounding the PSI core are arranged in two antenna belts. The four conserved LHCI subunits found in the PsaF/J side in plant and green algal PSI-LHCI complexes (*7*–*9*, *20*, *21*) are replaced by five LHCE subunits in *E. gracilis*. On the opposite side, where green algae typically only bind two LHCI subunits at the PsaB/M position, *E. gracilis* instead binds 11 LHCE subunits surrounding the entire PsaB/M/A side, forming a two-layer antenna arrangement (fig. S8).

The PSI-LHCE supercomplex exhibits a chimeric antenna composition as revealed by cryo-EM and sequence analysis (fig. S9). Its antenna system comprises a total of 16 subunits, specifically 12 Lhca proteins (LHCEs 1/2/6 to 13/15/16) and 4 Lhcbm proteins (LHCEs 3/4/5/14). Regarding the 12 Lhca proteins, although the amino acid sequences of LHCEs 1 and 2 are different, a segment of amino acid sequence from either Lhca10 or Lhca11 polyprotein could be modeled for both of these antenna proteins. On the basis of high-resolution amino acid density matching, LHCEs 6 and 9 are derived from distinct sequences segments within the Lhca7 polyprotein. In contrast, the full sequence of LHCE 8 originates from portions of both the Lhca5 and Lhca7 polyproteins. The amino acid sequences of LHCEs 7, 10, 12, 13, and 15 are all derived from specific segments of the Lhca6 polyprotein. However, a substantial portion of the amino acid sequences of LHCEs 7 and 10 is identical, while most amino acid sequences of LHCEs 12, 13, and 15 are highly conserved. LHCEs 11 and 16 are both derived from different parts of the Lhca5 polyprotein. Because of the relatively poor density of LHCE 16, its sequence was confirmed by matching amino acid side chains and conducting a BLAST analysis, which showed the closest match to sequences from the Lhca5 polyprotein. The naming of Lhca and Lhcbm follows that in *30*.

LHCEs 3 and 4 are derived from Lhcbm3 and Lhcbm1, respectively, and LHCEs 5 and 14 are derived from Lhcbm10*.* However, minor differences exist in their respective amino acid sequences (figs. S9 and S10). Phylogenetic analysis indicates that all LHCEs, including Lhca and Lhcbm proteins, are part of the Chl a/b–binding protein clade and form a monophyletic group, whereas Lhca proteins from the higher plant, moss, and green algae clustered together into another group (fig. S11). Moreover, the LHCEs diverged into two major clades. LHCE 3 to 5 and 14 clustered within one clade with *Arabidopsis* Lhcb2 (major LHCII) and Lhcb5 (CP26). In particular, LHCEs 3 and 4 exhibited a closer phylogenetic relationship to *Arabidopsis* Lhcb2 and Lhcb5 (fig. S11) (*45*).

All LHCE subunits have three major transmembrane helices (named αB, αC, and αA from N to C termini), two luminal amphiphilic helices (αE and αD), and three loop regions: an N-terminal loop, BC loop, and AC loop (fig. S12). The three transmembrane helices of individual LHCEs are relatively conserved, whereas the N/C-terminal loops and other loop regions exhibit flexibility (figs. S12 and S13). The structures of Lhcbm antenna proteins are highly conserved, except that LHCEs 3/4 have an additional short helix at the C terminus (fig. S13A). The structures of 12 Lhca proteins are also highly conserved, with the main differences lying in the lengths of their C-terminal loops and minor discrepancies in the CA loops (fig. S13B). *E. gracilis* Lhcbm and Lhca antenna proteins exhibit marked structural differences in the BC loop region and the C-terminal region. In particular, the BC loops in Lhcbm antenna are longer than those in Lhca proteins (fig. S13B). The structures of Lhca proteins in *E. gracilis* are relatively conserved compared to LHCIs in green algae (*7*, *8*) and plants (*19*, *45*). The structures of Lhcbm proteins in *E. gracilis* are highly conserved with those of major LHCII in green algae (*46*, *47*) and plants (*48*), as well as with the minor antenna CP26 in plants. In particular, LHCEs 3/4, plant CP26, and major LHCII show greater structural conservation, except for a slightly extended C terminus in LHCEs 3/4 (fig. S13, A, C, and D). However, there are notable differences in the BC loop of *E. gracilis* Lhcbm antenna and that of green algal CP26. There are also substantial disparities between *E. gracilis* Lhcbm proteins and CP29/CP24 in green algae or plants (fig. S13, E and F).

### Six pairs of LHCE heterodimers in PSI-LHCE

**Fig. 4.**
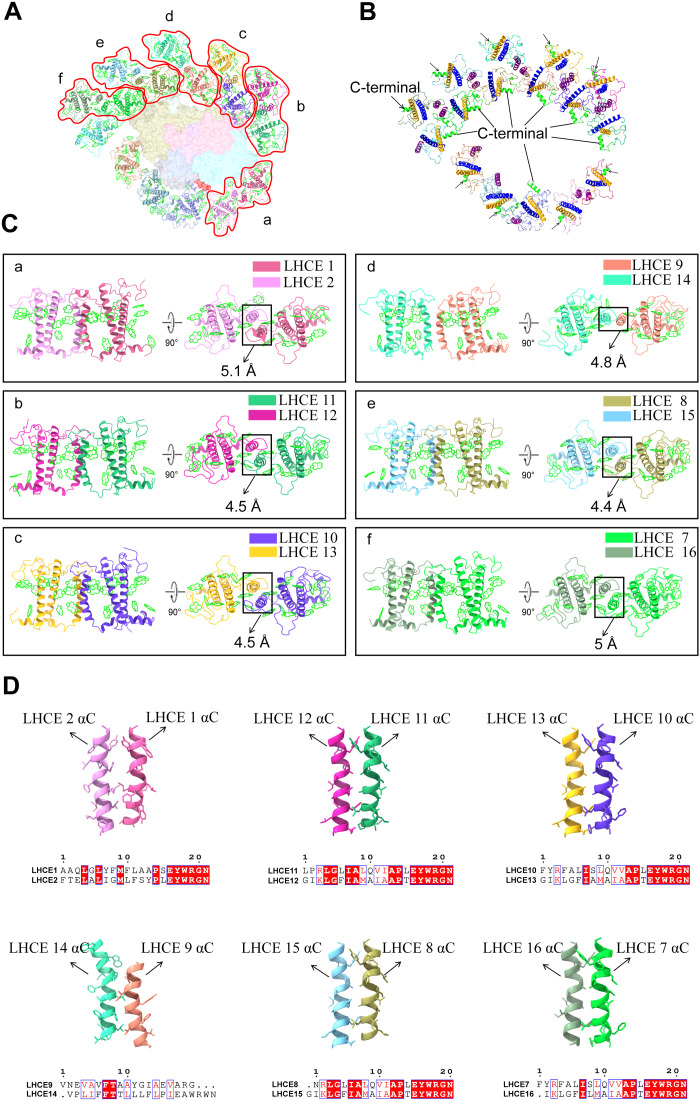
Locations and structures of LHCE heterodimers in PSI-LHCE. (**A**) Locations of six pairs of LHCE heterodimers in the PSI-LHCE supercomplex. (**B**) Spatial orientations of LHCE in *E. gracilis* PSI-LHCE. Color: Helices B, C, and A in LHCE are colored blue, magenta, and yellow, respectively. The C-terminal helix is in green. (**C**) Structures of heterodimers of LHCEs 1/2, 11/12, 10/13, 9/14, 8/15, and 7/16. (**D**) Parallelly arranged helix C and conserved amino acid sequences in the heterodimers.

The most prominent structural feature of the PSI-LHCE supercomplex is the presence of two layers of LHCE subunits on the PsaB/M/A side, resulting in six pairs of heterodimers that are entirely distinct from those found in other green algae and higher plants (Fig. 4A). All LHCEs have either undergone positional shifts or are located in positions completely different from the LHCI subunits in the red or green algal lineages (Fig. 4B and fig. S14). Firstly, the LHCE belt formed by LHCEs 1/2/3/4/5 occupies the positions corresponding to Lhca3-Lhca2-Lhca4-Lhca1, arranged from the PsaK to the PsaG side in higher plants. A key reason that more LHCEs can be accommodated in a comparable spatial position is that LHCEs 1 to 3 have experienced notable spatial orientation alterations (fig. S14). The spatial orientations of LHCEs 4 and 5 are similar to those of LHCIs in green algae and higher plants, with the C-terminal helices generally orienting toward the outer side of LHCI. However, LHCEs 1 and 3 have rotated about 50° to the left side relative to LHCE 4 observed from the stromal side, leading to a head-to-head, interdigitated arrangement of the helix B of neighboring LHCEs 3 and 4 at the stromal side. Notably, after undergoing a conformational flip, the additional helix at the C terminus of LHCE 3 is able to interact directly with PsaJ at the lumenal side. Even more intriguingly, LHCE 2 rotated nearly 180° to the right side in comparison to LHCE 4. Consequently, there is a substantial space between LHCEs 2 and 3, and LHCEs 2 and 1 form a distinct, closely packed heterodimer with a back-to-back arrangement of their C-helices (Fig. 4, B and C). The LHCE 1/2 heterodimer interface is stabilized through hydrogen bonding interactions between the AC loops of the two monomers on the stromal side (fig. S15). The interactions between this heterodimer and the PSI core are predominantly mediated by hydrogen bonds formed between the long N-terminal loop of LHCE 1 and the PsaA subunit on the stromal side, whereas hydrogen bonds are established through helix A of LHCE 1 and the BC loop of LHCE 2 on the lumenal side, both interacting with the PsaA subunit. Notably, LHCE 2 remains spatially distant from the PSI core on the stromal side and does not engage in any interactions with the core (fig. S16).

Five pairs of similar heterodimers are formed in the two-layer LHCE belt: LHCEs 7/16, 8/15, 9/14, 10/13, and 11/12 (Fig. 4, A and C). Unlike the parallel arrangement observed in the LHCE 1/2 heterodimer, each dimer consists of an LHCE from the inner layer and an LHCE from the outer layer, with a pair of closely spaced, parallel C-helices between its monomers. It is through the interactions between these two C-helices that the outer-layer LHCE subunits are bound to the inner-layer LHCE subunits (Fig. 4D). Notably, the structural orientations of all inner LHCEs (LHCEs 6 to 11) differ markedly from those of LHCEs 4/5 but resemble that of LHCE 3 (Fig. 4B). The C-terminal helices of LHCEs 6 to 11 are directed toward the interface between LHCEs and the core, and the C-helices of LHCEs 6 to 11 all point toward the outer region of the antenna. In contrast, the outer antennas (LHCEs 12 to 16) exhibit spatial orientations akin to those of LHCEs 4/5, with their C-helices oriented toward the inner antennas and their C-terminal helices pointing outward (Fig. 4B and fig. S14A). The distinctive, interlaced conformational arrangement of the inner and outer antennas positions all their C-helices in a “back-to-back” manner between the outer and inner antennas.

In the LHCE heterodimers, the two “back-to-back” C-helices contain a substantial number of hydrophobic amino acid residues. In addition, the terminal regions of the C-helices each contain a conserved amino acid motif: EYWRGN, which appears to facilitate their neat, parallel alignment (Fig. 4D). However, the two C-helices in LHCEs 9/14 are not perfectly aligned, likely due to alterations in their associated hydrophobic amino acid residues. LHCEs 9/14 lack the complete conserved amino acid motif but still retain key amino acid residues such as E•WR. Thus, the key sequence (E•WR••) may be crucial for the close, parallel arrangement of the adjacent C-helices and may therefore also be essential for the formation of the heterodimers in *E. gracilis*.

Interactions between the inner LHCE belt (LHCEs 6 to 11) and the PSI core are primarily mediated by hydrogen bonds and hydrophobic interactions between LHCE 8, LHCE 10, and the core subunits (fig. S16), while no substantial interactions are observed between the other LHCEs and the core. On the lumenal side, these interactions are established through hydrophobic helix D at the C termini of LHCEs 8/10, which form hydrogen bonds with PsaM and PsaA, respectively. Notably, on the stromal side, the helix A of LHCE 10 engages in prominent hydrogen bonding with two additional helices at the N terminus of PsaD, which are located at the membrane surface of the stromal side and stabilize the binding of LHCE 10 to the PSI core.

Consequently, the deletion of key core subunits (e.g., PsaG/I/L/H/O/K/N) from PSI-LHCE may have triggered a marked structural rearrangement. The anchor points for LHCE are supplied directly by PsaA/B/D/F/J/M in the resulting structure, bypassing the need for the lost subunits. Moreover, this reorganization leads to the formation of six tightly packed LHCE heterodimers—a structural innovation that markedly differentiates *E. gracilis* from its green algal relatives and underscores its unique adaptations in the light-harvesting machinery.

### Lhcbm protein structures and their special pigment compositions

In addition to the six heterodimeric pairs mentioned above, three Lhcbm proteins (LHCEs 3, 4, and 5) and one Lhca protein (LHCE 6) are present on the PsaB/F side. LHCEs 3, 4, and 5 are adjacent to one another, but they do not form heterodimers. A conformational flip of LHCE 3 introduces multiple hydrogen-bonding interactions between its helix B/C and the N-terminal loop of LHCE 4 at the stromal side, suggesting a potential dimerization between LHCEs 3 and 4 (fig. S15B). LHCEs 3, 4, and 5 are spatially distant from adjacent LHCEs 2 and 6, with a particularly large gap between LHCEs 6 and 5. In addition, the low electron density map observed for LHCE 6 indicates weak binding of LHCE 6 to PSI.

**Fig. 5.**
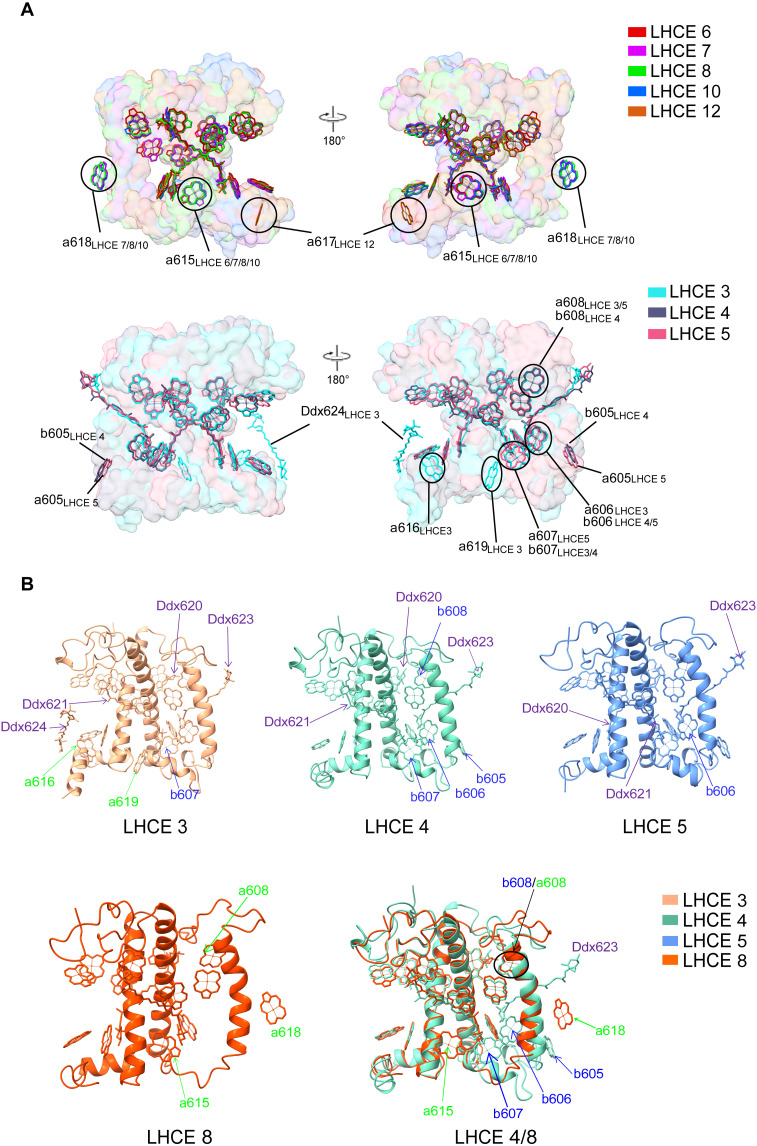
Structure and pigment-binding sites of LHCE proteins. (**A**) Superposition of pigments of Lhca and Lhcbm proteins. The special pigments are circled and labeled. (**B**) Structure and pigment-binding sites of LHCEs 3, 4, 5, and 8 and comparison of pigments among them.

Each LHCE binds 8 to 16 Chls and 1 to 4 Ddx molecules (table S2), and there are pronounced differences in the bound pigments between the Lhca and Lhcbm proteins, particularly regarding their ability to bind Chl b on the basis of our high-resolution map. All Lhca antenna proteins bind only Chl a but no Chl b. The Lhcbm antenna can bind one Chl b, but the binding site for Chl b is not conserved. In contrast, LHCE 4 can bind four Chls b, making it the antenna with the lowest ratio of Chl a to Chl b in *E. gracilis* (Fig. 5 and table S4). Moreover, the total number of Chls (14 to 1) and Ddx (3 to 4) in each antenna of LHCEs 3/4/5 are higher than those in Lhca proteins (8 to 13 Chls and 1 to 3 Ddx). LHCE 3 binds the largest number of Chls (14 Chls a and 1 Chl b) and Cars (4 Ddx), whereas LHCE 4 binds the largest number of Chls b (Fig. 5B). These Chl b binding sites in LHCE 4 are conserved in the higher plant LHCI (fig. S17) (*19*). LHCEs 3 and 4 each bind 1 Chl b at positions 607 and 606, respectively, whereas all other Chl binding sites are occupied by Chl a. In addition, LHCE 14 is also a Lhcbm protein. However, because of the poor density, it is impossible to determine whether Chl 605 to 608 molecules are Chl a or b in LHCE 14.

**Fig. 6.**
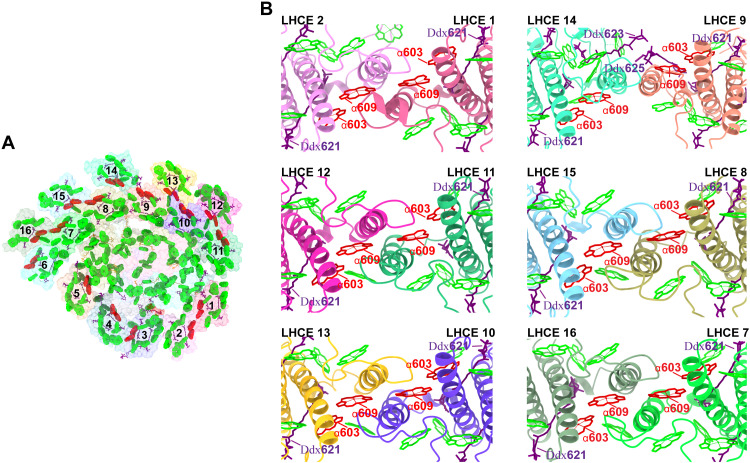
Arrangement of Chl a603-a609 in PSI-LHCE. (**A**) Chl a603-a609 in close proximity within the heterodimer. (**B**) Chl a603-a609 pairs bound to LHCEs 1/2, 9/14, 11/12, 8/15, 10/13, and 7/16.

The binding positions of most Chls and Cars in LHCEs are conserved in LHCIs of higher plants (*19*) and green algae (fig. S17) (*7*, *8*), and there are only differences in some pigment binding sites among LHCEs (Fig. 5 and table S4). The conserved Chl a binding sites, 601 to 604 and 608 to 614, are present in all LHCEs, with the following exceptions: LHCE 14 lacks Chl a601, LHCE 4 binds Chl b instead of Chl a at 608, LHCEs 14 to 16 lack Chl a611, and LHCEs 1/2/13/14/16 lack Chl a614.

The substantial variations in the Chl binding sites between Lhcbm and Lhca proteins are located at 605, 606, 607, and 608 sites near the lumenal side, which are capable of binding Chl b (Fig. 5). LHCEs 3/4/5/14 all bind Chl at these sites, but the Lhca proteins do not bind any pigments at 605/606/607, except that LHCE 2 binds Chl a near the 605 site, and LHCE 3 does not bind a pigment at this 605 position. Chl b occupies the Chl 606 site in LHCEs 4/5 and the 607 site in LHCEs 3/4, which is conserved in higher plant LHCI (*19*). However, the 606 site in LHCEs 3/14 and the 607 site in LHCEs 5/14 are occupied by Chl a instead of Chl b. LHCEs 6 to 11 have a unique Chl a615, which is located in the gap between the inner- and outer-layer antennas on the lumenal side and is not found in other antenna proteins, suggesting its role in capturing and transferring energy between the inner and outer antenna layers. Moreover, each of LHCEs 7/8/10/11 contains a special Chl a618, which is positioned near the helix C of these heterodimers, suggesting its potential involvement in energy transfer between the outer and inner antennas (Fig. 5).

Notably, LHCE 3 has two unique Chls a (named Chls a616 and a619) on the lumenal side (Fig. 5B). Among them, Chl a619 is absent in other LHCEs as well as in the LHCI of higher plants and green algae. The position of Chl a616 in LHCE 3 is similar to that in *Chlamydomonas*, but the porphyrin ring planes of these two Chl a molecules are slightly tilted with respect to one another. Chl a619 is adjacent to Chl a615 in LHCEs 6 to 11, but the porphyrin ring of Chl a619 (in LHCE 3) is nearly perpendicular to that of Chl a615 (in LHCEs 6 to 11). Chl a619 is located between LHCEs 3 and 4 on the lumenal side and is in close proximity to the Chl a613 of LHCE 4. The two Chls may therefore be involved in energy transfer between LHCEs 3 and 4. The Chl a616 of LHCE 3 is located between LHCE 3 and PsaJ, with a special Ddx624 molecule adjacent to it. Moreover, Chl a616 and Ddx624 are very close to Ddx in the core subunit. Therefore, Chl a616 may be involved in the energy transfer between LHCE 3 and the core, and Ddx624 and Ddx on the core subunit may play a role in photoprotection.

All LHCEs in *E. gracilis* bind only Ddx as their Cars, instead of Lut, violaxanthin, or β-Car in higher plants (*19*) and green algae (tables S2 and S4) (*7*, *8*). LHCEs 4/5/14 each bind three Ddx, whereas only LHCE 3 binds four Ddx. In contrast, most Lhca proteins bind two Ddx, except LHCE 9 with three Ddx and LHCE 16 with only one Ddx, which may partially be caused by the poor density of LHCE 16. The positions of three Ddx (620, 621, and 623) in LHCEs 3/4/5/14 are highly similar to L1, L2, and N1 sites in higher plant and green algal LHCIs (fig. S17). However, the additional Ddx 624 of LHCE 3 is not conserved with Lut 624 in higher plant Lhca1. Lut 624 is located between Lhca1 and Lhca4, whereas Ddx 624 is located between LHCE 3 and PsaA. The head of Ddx 624 is very close to Chl a793 on PsaA (3.16 Å), indicating that the function of this Car may be energy transfer between LHCE 3 and the core. In most Lhca proteins, there are two conserved Ddx-binding sites: Ddx 620 and Ddx 621. However, LHCE 9 also contains an additional Ddx 625, which is not conserved with that of Ddx 624 in LHCE 3 (fig. S17). Ddx 625 is located between helices B and C of LHCE 9, as well as between the two C-helices of the LHCE 9/14 heterodimer, and is very close to the Ddx 623 of LHCE 14.

### Special Chl a603-a609 pairs in the heterodimer

To enhance photon capture, higher plants’ PSI uses long-wavelength Chls to absorb far-red light. This far-red absorption capability directly originates from the tightly clustered Chl pair within the Lhca3 and Lhca4 subunits of the LHCI antenna, known as the “red Chls,” which includes the Chl a603-a609 dimer (*9*, *19*–*21*, *49*). In the *E. gracilis* PSI-LHCE supercomplex, each LHCE contains the conserved Chl a603-a609 pair (Fig. 6A). However, because of changes in the spatial orientations of most LHCEs, the Chl a603-a609 pairs are not located between the antenna and the core as they are in higher plant PSI. For example, Chl a603-a609 in LHCE 2 is located on the outer surface of the LHCE 2 antenna subunit, Chl a603-a609 in LHCE 3 is located between LHCEs 3 and 4, and Chl a603-a609 pairs in the inner and outer layers of the PsaB/M/A LHCE belts are all located on helix C between the inner and outer antennas.

These Chl a603-a609 pairs form a tightly clustered group, situated in the intermembrane space between LHCI layers or at the interface of adjacent LHCIs. The coordinated amino acid residues of Chl a603-a609 in *E. gracilis* are highly conserved in higher plants (fig. S18) (*19*). However, because of the parallel and tight arrangement of these back-to-back C-helices, the distances between the two pairs of Chl a603-a609 in the heterodimers are very short. The two pairs of Chl a603-a609 in LHCEs 1/2, 7/16, 8/15, 9/14, 10/13, and 11/12 exhibit a parallelogram-like arrangement, with the Chl a609-a609 distance being even shorter (Fig. 6B). Moreover, two or three Ddx are always bound around Chl a603-a609, suggesting that these Chl pairs may play an important role in the interantenna energy transfer process and energy dissipation under high light environment.

### Energy transfer in the PSI-LHCE supercomplex

**Fig. 7.**
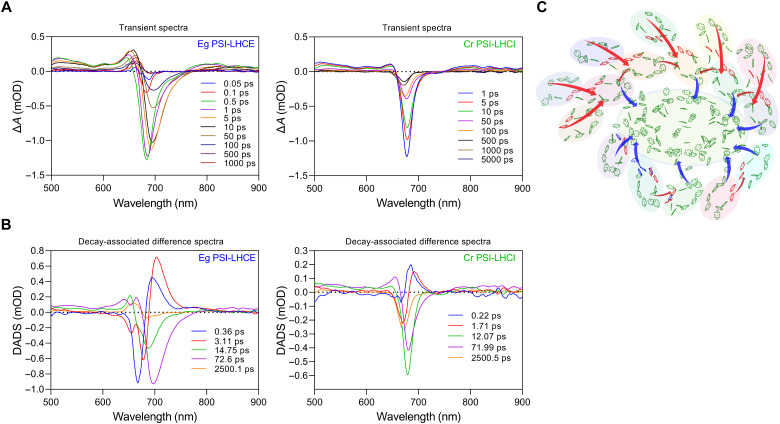
Exciton energy transfer dynamics and pathways in the *E. gracilis* PSI-LHCE supercomplex. (**A**) Femtosecond transient absorption spectra of PSI-LHCI from *E. gracilis* and *C. reinhardtii*, with excitation at 670 nm. (**B**) Decay-associated difference spectra (DADS) and corresponding decay components for PSI-LHCI from *E. gracilis* and *C. reinhardtii.* (**C**) Potential exciton energy transfer pathways within *E. gracilis* PSI-LHCE. Standard Chls a are colored green, while the specific Chl a603-a609 is colored red, and Chl b is colored blue. Blue arrows depict energy transfer from the peripheral antenna system to the PSI core. Red arrows indicate energy transfer pathways between individual antenna units (LHCE).

On the basis of the high-resolution density map, 330 pigment molecules are identified in the PSI-LHCE supercomplex (table S2). Using Förster resonance energy transfer (FRET) calculations, we computed the pairwise FRET rate constants (*k*_FRET_) for intersubunit exciton energy transfer pathways (fig. S19 and tables S5 and S6) on the basis of the structural configuration, illustrating potential rapid energy transfer pathways. To examine the energy transfer efficiency of the PSI-LHCE complex from *E. gracilis*, we carried out femtosecond transient absorption spectroscopy under annihilation-free conditions, selectively exciting the Chls at 670 nm, and used the PSI-LHCI complex from *Chlamydomonas reinhardtii* for comparison (Fig. 7A). The excited-state absorption and ground-state bleaching dynamics of *Euglena* PSI-LHCE were similar to those of its *Chlamydomonas* counterpart, which can be seen more clearly from the global fitting results with a parallel model (Fig. 7B). All kinetics profiles were well fitted using a parallel model with five characteristic kinetic decay components, spanning from 0.36 ps to 2.5 ns for *E. gracilis* and 0.22 ps to 2.5 ns for *Chlamydomonas*. The fastest decay component that occurred at <1.0 ps likely represents energy transfer inside the same coherent domain and/or vibrational relaxation processes. The second decay component occurs within a few picoseconds. In this decay-associated spectrum, the positive peak is at >700 nm for *E. gracilis*, which is more red shifted than the corresponding component in *Chlamydomonas* (<700 nm). This red-shifted component in *E. gracilis* may reflect a large-scale energy transfer from bulk Chls to red Chls within the antenna. Components around several tens of picoseconds (15 and 72 ps) represent the energy equilibrium within the supercomplex, while a nanosecond-lived component (≈2.5 ns), exhibiting a blue-shifted decay-associated spectrum, may be attributed to uncoupled LHCEs or detached Chls that are not kinetically coupled to the primary PSI-LHCI complex (*50*).

The main component representing the overall excitation trapping time for energy transfer from the antenna to the core was found to be ~72 ps (a dominant kinetic decay component) for both *E. gracilis* PSI-LHCE and *Chlamydomonas* PSI-LHCI. This substantially short trapping time indicates that despite the antenna size of *E. gracilis* PSI-LHCE being larger than that of the *Chlamydomonas* PSI-LHCI supercomplex, its photosynthetic quantum efficiency remains as high as 96.5%, on par with *Chlamydomonas* (*50*, *51*).

## DISCUSSION

**Fig. 8.**
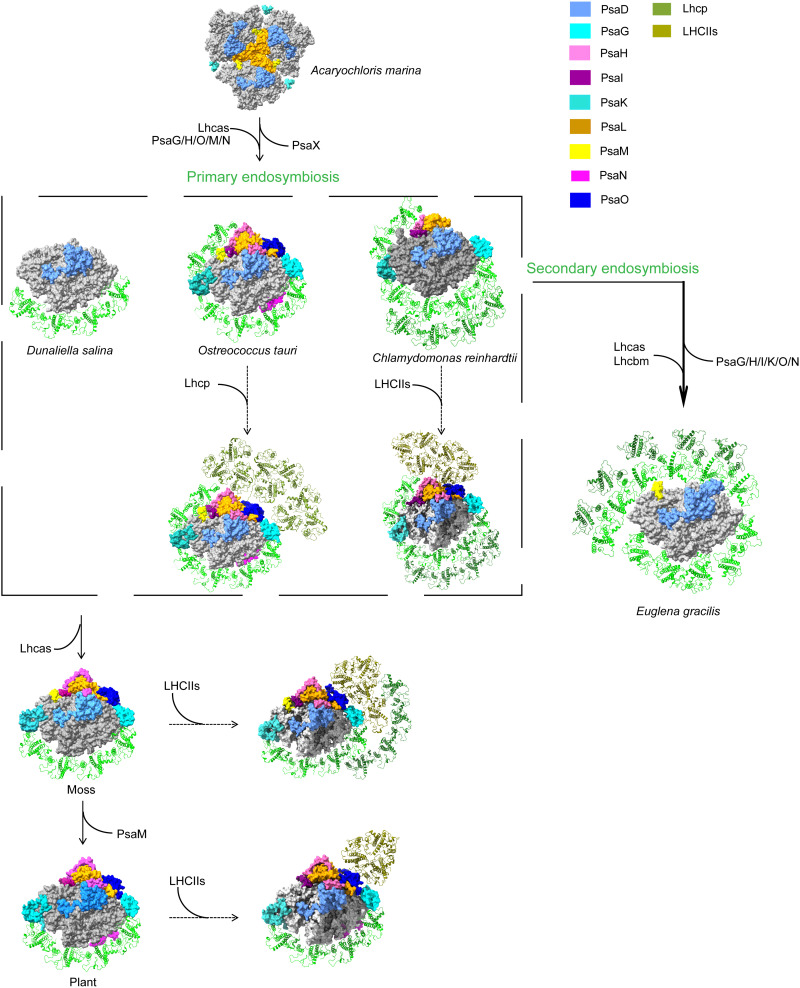
Possible evolutionary changes of PSI-antennae from the cyanobacteria to the green lineages. PDB codes of PSI structures: PSI of a cyanobacterium (*Acaryochloris marina*, PDB code: 7COY), PSI-LHCI of green algae (*D. salina* PSI-LHCI, PDB code: 6YXR; *O. tauri* PSI-LHCI and PSI-LHCI-Lhcp, PDB code: 7YCA; *C. reinhardtii* PSI-LHCI, PDB code: 6IJO; *C. reinhardtii* PSI-LHCI-LHCII, PDB code: 7D0J), a moss (*P. patens* PSI-LHCI and PSI-LHCI-LHCII, PDB code: 7XQP), a higher plant (*Zea mays* PSI-LHCI and PSI-LHCI-LHCII, PDB code: 5ZJI), and *E. gracilis* in this study. Special core subunits are differentiated by different colors.

The ability of oxygen-evolving photosynthetic organisms to adapt to different environmental conditions holds substantial evolutionary and ecological significance. Here, we show that the structure of PSI-LHCE from secondary endosymbiotic green flagellate *Euglena* differs greatly from that of primary endosymbiotic green algae, mosses, and plants (Fig. 8). Compared to its counterparts in green algae and plants, *E. gracilis* has a more compact PSI core (eight subunits) and a larger antenna absorption cross section. It lacks the PsaG, PsaH, and PsaN subunits shared by other green algae and higher plants (*7*–*21*), as well as the PsaI, PsaL, PsaO, and PsaK subunits common to both green- and red-lineage organisms (*4*–*6*, *23*–*26*). In plants, mosses, and green algae, PsaG and PsaK serve as crucial connection points between LHCI and the PSI core. In higher plants, PsaG is linked to Lhca1, while PsaK binds to Lhca3 (*16*, *19*, *52*, *53*). In addition to serving as an anchor for LHCI binding, PsaG also plays a role in stabilizing the PSI core (*54*), and PsaK is involved in the function and organization of the LHCI to the PSI core (*53*). In addition, PsaK participates in the state transition processes in higher plants, bryophytes, and green algae (*53*, *54*). Recent structural biology evidence showed that in the PSI-LHCI-Lhcp supercomplex of prasinophyte *Ostreococcus tauri*, PsaK directly binds to an Lhcp trimer (*11*). In contrast, in the PSI-LHCI-LHCII supercomplex of *Physcomitrium patens*, PsaK interacts with the moss-specific LHC protein Lhcb9, which appears to function in integrating the entire antenna complex (*14*, *15*). Although PsaG is lost in the red-lineage algae, most of them have evolved PsaR that replaces PsaG and connects to their respective LHCIs (*6*, *23*, *40*). The spatial positions originally occupied by PsaG/I/K are vacant in *E. gracilis* (even though the subunits themselves are absent). In the structure of *E. gracilis* PSI-LHCE, there is also an absence of the PsaH/L/O subunits. These subunits are well known as the docking sites for LHCII trimers during state transitions in higher plants, mosses, and green algae (*9*, *15*, *46*, *55*, *56*). Furthermore, the lack of PsaH facilitates some degree of PSI dimerization in *Chlamydomonas* (*57*). Near the PsaH/L subunit sites, *E. gracilis* has evolved LHCEs 9 and 10, which occupy part of the space that would otherwise be filled by PsaH/L. Similarly, LHCE 11 occupies portions of the PsaO and PsaK positions. The absence of these core subunits (PsaG/I/H/L/K/O) results in a more compact PSI core that is surrounded by a larger number of LHCEs. Given that PsaH, PsaL, and PsaO are the known docking sites for LHCII during state transitions in green algae and plants, our PSI-LHCE structure suggests that if state transitions do occur in *E. gracilis*, the binding site for LHCII and its association mechanism with PSI are likely to be fundamentally different from those found in plants and other green algae.

*E. gracilis* PSI contains fewer core subunits but binds a large number of LHCEs, indicating that the loss of core subunits does not necessarily result in a decrease in the number of LHCEs. Moreover, despite the absence of the conserved peripheral core subunits (PsaG/I/K) present in higher plants and green algae, their original specific positions are not only preserved in *E. gracilis* but also remain unoccupied and unsubstituted by other protein subunits (fig. S20). Therefore, we propose that the binding between the LHCE antenna and core modules is not only mediated by key core subunits as previously assumed. Instead, adjacent LHCEs can first form tight interactions among themselves. Subsequently, specific LHCEs establish anchor sites with core subunits, ultimately enabling the entire LHCE module to connect to the core and facilitating energy transfer. We observed that many antenna proteins in *E. gracilis* PSI-LHCE exhibited altered spatial conformations, leading to the formation of tightly packed heterodimers between adjacent antennas and most inner-to-outer layer antennas. We propose that the back-to-back arrangement of transmembrane helices in the LHCE heterodimers, the conserved interaction motifs, and the conformational changes in the LHCE allow them to form tight connections with each other. These strong antenna-antenna connections appear to be the primary driving factor for the multitude of LHCE subunits to form a supercomplex with the compact core subunits.

The structural arrangement of PSI-LHCE and the location and pigment compositions of antenna proteins in *E. gracilis* exhibit substantial evolutionary features. The structure of *E. gracilis* PSI-LHCE shares common features with typical green-lineage organisms. For instance, the positions and quantities of most core subunits and pigments are highly conserved between *Euglena* and green-lineage organisms (fig. S5). However, this alga has evolved unique structural arrangements and pigment features, exhibiting a red-green hybrid structural characteristic. Specifically, we have found not only a large number of LHCEs in *E. gracilis* but also that these LHCEs can assemble into closely spaced dimers, which may be a result of the secondary endosymbiosis of the *E. gracilis* plastid, reflecting a special binding mode of antenna proteins. In terms of pigment composition, *E. gracilis* also displays a red-green hybrid feature. It contains both the conserved Chl a/b in green lineage organisms and the Ddx commonly found in red-lineage organisms. The binding sites of these Ddx in LHCE are highly conserved with those of Cars in higher plant LHCI, although the types of pigments bound have changed. The four Ddx and PsaM/β-Car bound to the PSI core exhibit characteristics similar to those in diatoms and other red-lineage organisms. These structural and pigment observations highlight the evolutionary plasticity of photosynthetic systems. This finding is further corroborated by the recent work of Kato *et al.* (*58*), who described the PSI structure of *E. gracilis* strain Z with 13 LHCI subunits. Their phylogenetic analysis also suggested that the nuclear-encoded core subunit PsaD likely originated from cyanobacteria via lateral gene transfer. Together, these observations underscore the mosaic origin of the *Euglena* PSI-LHCI, revealing the highly plastic nature of photosynthetic machineries during their long-term adaptation and evolution.

Our structural study also revealed that the light-harvesting antenna of PSI in *E. gracilis* exhibits considerable heterogeneity. Its PSI core binds not only Lhca proteins but also Lhcbm proteins. Notably, Lhcbm and Lhca antenna proteins not only show distinct structural differences in the BC-loop region and the C-terminal region, but these two types of antenna proteins also exhibit completely different affinities for Chl b. Lhcbm proteins can bind Chls a and b, while Lhca proteins only bind Chl a, showing a marked difference from primary endosymbiotic green-lineage organisms. Moreover, the amount of Chl b in Lhcbm proteins encoded by different genes also varies: the Chl a/b ratio of LHCE 4 is 2.5, whereas the Chl a/b ratios of LHCEs 3 and 5 are 14:1 and 13:1, respectively. This indicates that during the secondary endosymbiosis of green-lineage organisms, Lhca proteins have nearly lost the ability to bind Chl b, whereas Lhcbm proteins still retain a Chl b–binding capacity similar to that in green algae and plants, although there are differences in the amount of Chl b bound by different Lhcbm proteins.

In summary, the 2.23-Å resolution cryo-EM structure of PSI-LHCE in *E. gracilis*, an organism from the green lineage that underwent secondary endosymbiosis, provides key insights into the evolution of green photosynthetic organisms. Compared to its counterpart in higher plants and green algae, the *E. gracilis* PSI-LHCE complex exhibits unique structural and compositional features, including its protein subunit organization, antenna size and spatial conformational changes, and pigment composition. Ultimately, these findings provide key insights into the molecular mechanisms of *Euglena*’s environmental adaptation and greatly contribute to our understanding of the evolutionary process of photosynthetic organisms and how algae optimize their protein structure and pigment networks to adapt to their respective living environments.

## MATERIALS AND METHODS

### Purification of PSI-LHCE from *E. gracilis*

*E. gracilis* (catalog number: FACHB-848) was purchased from Freshwater Algae Culture Collection at the Institute of Hydrobiology (FACHB), National Aquatic Biological Resource Center. To verify the strain, we performed 18*S* ribosomal RNA and internal transcribed spacer (ITS) gene sequencing, confirming that the strain belongs to *E. gracilis*. The primers 18s-ITS-F “5′-TTCAGCTTCTCTGAGGTGCTGTG-3′” and 28s-ITS-R “5′-TCGGTGTGGGGGTTCTGTATCGA-3′” were used for the identification of *E. gracilis*.

*E. gracilis* cells were cultured in 10 liters of HUT medium and were grown with constant stirring and air bubbling under continuous white light (35 μmol photons m^−2^ s^−1^) at 25°C for about 1 week until they reach absorbance of 1.3 optical density at 730 nm. Cells were collected using centrifugation (6000*g* for 10 min, R9A2-4608) and were washed once with an HMCS buffer (25 mM Hepes-KOH, pH 7.5, 5 mM MgCl_2_, 5 mM CaCl_2_, and 0.33 M sucrose). The cells were resuspended in 50 ml of the HMCS buffer and then broken by a pressure cell of Avestin EmulsiFlex-C3 (two cycles at 27.6 Mpa). The lysate was cleared by centrifugation using a P40ST rotor in an ultracentrifuge CP100NX (Hitachi, Japan) for 30 min at 65,000*g*. Membranes in the supernatant were pelleted using ultracentrifugation (R20A2-5023, 40,000*g* for 30 min), washed with 50 ml of HMCE buffer (25 mM Hepes-KOH, 5 mM MgCl_2_, 5 mM CaCl_2_, and 5 mM EDTA), and resuspended in an HB buffer (20 mM Hepes-KOH and 1 M betaine, pH 7.5).

The thylakoids were solubilized with 0.8% (w/v) *n*-dodecyl-β-D-maltoside (β-DDM) at a Chl concentration of 0.4 mg ml^−1^ for 20 min on ice in the dark with gentle stirring. After centrifugation at 21,000*g* for 20 min at 4°C, the resultant supernatant was loaded onto a linear sucrose density gradient of 5 to 30% (w/v) sucrose in a medium containing 25 mM Hepes-KOH (pH 7.5), 1 M betaine, 10 mM CaCl_2_, 10 mM MgCl_2_, and 0.02% β-DDM. After centrifugation at 243,000*g* for 18 hours at 4°C (P40ST rotor; Hitachi), the PSI-LHCE band was collected and further purified by gel filtration chromatography (Superose 6 Increase 10/300 GL) in an HBN buffer (25 mM Hepes-KOH, 0.5 M betaine, 50 mM NaCl, and 0.02% β-DDM). The sample was then concentrated using a 100-kDa cutoff filter (Amicon Ultra; Millipore) by centrifugation at 4000*g*.

### Characterization of the PSI-LHCE supercomplex

Subunit composition of *E. gracilis* PSI-LHCE was analyzed by a 16 to 22% SDS–polyacrylamide gel electrophoresis (PAGE) gel containing 7.5 M urea as described previously (*59*). *E. gracilis* PSI-LHCE corresponding to 2 μg of Chl was solubilized for 10 min at 60°C after adding 2% lithium lauryl sulfate and 75 mM dithiothreitol. A standard molecular weight marker (C610013; BBI) was used. The separated subunit bands were identified by mass spectrometric analysis.

Ultraviolet absorption spectra were recorded by a ultraviolet-visible spectrophotometer (U-3900H, Hitachi, Japan) at room temperature. The fluorescence emission spectra were measured at 77 K with a fluorescence spectrometer (F-7100, Hitachi, Japan) equipped with a xenon lamp, and the spectra were recorded at a wavelength range from 600 to 800 nm with an excitation wavelength of 436 nm.

### Pigment composition

Pigment composition was analyzed using a Waters e2695 high-performance liquid chromatography system equipped with a C18 reversed-phase column (4.6 by 250 mm, 5-μm particle size, Grace, US). The pigments were extracted in 80% (v/v) cold acetone from purified *E. gracilis* PSI-LHCE, and the elution was performed as described previously (*23*). The pigments were detected by their absorbance at 445 nm with a wavelength detection range of 300 to 800 nm. Pigments were identified on the basis of their retention times and absorption spectra based on those of authentic standards (*37*, *38*, *60*).

### Femtosecond transient absorption spectroscopy measurements

For the ultrafast experiment, the HARPIA-TA transient absorption spectroscopy system (Light Conversion) was used. The pump pulse centered at 670 nm was generated through a noncollinear optical parametric amplifier (ORPHEUS-N-2H, Light Conversion) pumped by the double frequency of the 1030-nm femtosecond laser (PHAROS, Light Conversion) at a 25-kHz repetition rate. The broadband probe pulse covering 500 to 900 nm was generated by focusing the 1030-nm femtosecond pulse into a sapphire plate, with a postmeasurement chirp correction. The excitation intensity was 2 nJ per pulse. The sample absorbance was kept with OD (optical density) = ~0.4 at 670 nm in a 2-mm cell. To prevent photodamage, the sample was continuously stirred during measurement. Global fitting was conducted with CarpetView software (*61*).

### Cryo-EM sample preparation and data collection

An aliquot of 3.0 μl of purified PSI-LHCE was applied to a holey carbon grid covered with a graphene oxide film (Quantifoil R1.2/1.3, Au, 300 mesh), left for 5 s, blotted for 4 s at a humidity of 100% and 4°C, and plunged into precold liquid ethane in a Vitrobot (FEI). Cryo-EM images were collected on a Titan Krios microscope (FEI) operated at 300 kV equipped with a Gatan Quantum energy filter (with a slit width of 20 eV) and a K3 camera (Gatan) operated at the super resolution mode, with a magnification of 81,000. Each movie comprises 32 frames with a total dose of ~60 e/Å^−2^, an exposure time of 1.8 s, and a dose rate of 39 e^−^ pixel^−1^ s^−1^. Data acquisition was carried out using EPU software (Thermo Fisher Scientific) with a defocus range of −1.0 to −2.0 μm. The final images were binned, resulting in a pixel size of 1.04 Å, for further data processing.

### Cryo-EM image processing

All movie stacks were corrected by MotionCor2.136 with dose weighting. CTF parameters for each movie were estimated by CTFFIND-437. Image processing was accomplished with CryoSPARC version 4.2 (*62*). We captured a total of 22,430 micrographs and selected 5,138,736 particles automatically. After several rounds of selection through two-dimensional classification, 562,173 particles were selected for ab initio reconstruction. Subsequently, heterogeneous refinement was performed, which yielded three classes of particles. We selected a class containing 305,958 particles and further subjected it to nonuniform refinement, which resulted in a structure with a gold-standard Fourier shell correlation resolution of 2.23 Å. To improve the resolution of the cryo-EM density maps, we further refined their densities with local refinement using local masks, resulting in final resolutions of 2.63 Å for the peripheral antennae.

### Mass spectroscopy analysis

Purified PSI-LHCE was dissociated and run on an SDS polyacrylamide gel. The gel was briefly stained with Coomassie Brilliant Blue and analyzed using mass spectrometry. The protein bands visualized by Coomassie Brilliant Blue staining were excised from the SDS-PAGE gel for mass spectrometric identification. Following in-gel digestion with sequencing-grade modified trypsin, the resulting peptides were extracted. Peptide separation was carried out on an EASY-nLC 1000 system using a 90-min linear gradient at a flow rate of 300 nl/min. The eluent was directly ionized and analyzed with a Thermo Orbitrap Fusion mass spectrometer. All tandem mass spectrometry spectra were searched against a database using Proteome Discoverer software (version 1.4.0.288).

The Ddx fraction, collected after liquid chromatographic elution, was subjected to molecular weight identification using an ultrahigh-performance liquid chromatography system (Vanquish Neo, Thermo Fisher Scientific, US) coupled to a high-resolution mass spectrometer (Orbitrap Exploris 480, Thermo Fisher Scientific, US). The analysis was performed with a scan range of *m*/*z* (mass/charge ratio) 300 to 2000, a maximum injection time of 100 ms, and an Orbitrap resolution of 15,000.

### Model building and refinement

As the genomic sequence for *E. gracilis* has only a preliminary sequence annotation, and the UniProt database did not cover the sequence of all proteins, we obtained the protein sequences of PsaA/B/C/M/J only from the *E. gracilis* database when constructing the structures of the core subunits. Moreover, the sequences for PsaD/E/F subunits were either not available or incomplete in the database. Therefore, we used the corresponding sequences from *C. reinhardtii* [Protein Data Bank (PDB) code: 6IJO] as templates to guide the model building of PsaE/F subunits. Specifically, the sequence of PsaD was initially fitted into the density map on the basis of the PsaD sequence from *C. reinhardtii*, but some additional electron density was observed. To address this issue, amino acid sequences were manually deduced by identifying amino acid side chains in the clear electron density map.

The structural construction of *E. gracilis* LHCE was carried out on the basis of the antenna protein sequences from the UniProt database combined with mass spectrometric data. Given that the primary *Lhc* genes in *E. gracilis* are organized as contiguous coding regions (concatenated Lhc sequences), which are transcribed and translated into large, single polyproteins encompassing both Lhca and Lhcb/Lhcbm proteins (*33*), and AlphaFold3 (*63*) prediction results suggest that the antenna proteins may form multimers, we first separated these multimers into monomers and then used AlphaFold3 to conduct structural predictions again. Ultimately, the monomer antenna subunits were used for the model construction of LHCE. Notably, the sequences used for LHCEs 3 and 4 were derived from Lhcbm3 and Lhcbm1, respectively, while the sequences for LHCEs 5 and 14 were derived from Lhcbm10. This was because when these four antenna proteins were constructed using Lhca monomers, there was a mismatch in amino acid densities, and the amino acid sequences were not long enough to cover the density map.

All protein subunits were initially fitted into the cryo-EM density map using Chimera X1.9 (*64*, *65*) and subsequently refined manually with Coot (*66*). The model was then subjected to stereochemical refinement using phenix.real_space_refine in the PHENIX suite (*67*). The final model was visualized using PyMOL, UCSF Chimera 1.19 (*64*), and Chimera X (*68*). Local resolution maps were generated with CryoSPARC version 4.2, while all structural figures were prepared using PyMOL and Chimera.

### Phylogenetic analysis

With the exception of *E. gracilis*, all LHC protein sequences were retrieved from the UniProt database. For phylogenetic tree construction, we first aligned sequences with MUSCLE using default parameters and then generated the tree topology using MEGA X (*69*). The neighbor-joining method was applied to infer phylogenetic relationships (*70*). Branch labels indicate bootstrap support percentages from 1000 replicates (*71*). Distance matrices were generated using Poisson-corrected evolutionary distances (*72*).

### Computational analysis of FRET in PSI-LHCE

The final atomic model was utilized for FRET calculations based on Förster theory (*73*, *74*). The Förster energy transfer rate constants was computed as *k*_FRET_ *=* (*C*κ^2^)/(*n*^4^*R*^6^), where *k*_FRET_ represents the energy transfer rate, *C* is a factor calculated from the overlap integral between the two Chls, κ is the dipole orientation factor, *n* is the refractive index, and *R* is the distance between two central magnesium atoms of Chls. The FRET rates were computationally calculated using a custom-made algorithm on the Python platform (Python version 3.9) and visualized in Chimera X (*65*, *68*). The characteristic pairwise transfer time (τ_transfer_) is calculated by the inverse of the rate constant: τ_transfer_ = 1/*k*_FRET_.
