# Supplementary material for: Structural basis of diadinoxanthin-Chl a/b–binding proteins in the photosystem I supercomplex of Euglena gracilis
Source: Sci Adv. 2026 Mar 6;12(10):eaea5561. doi: 10.1126/sciadv.aea5561 (PMC12965295; doi:10.1126/sciadv.aea5561)
Supplement: Supplementary file 1 — Figs. S1 to S20 Tables S1 to S6 [file sciadv.aea5561_sm.pdf]

Science Advances
AAAS

Supplementary Materials for

Structural basis of diadinoxanthin-Chl a/b–binding proteins in the photosystem I supercomplex of *Euglena gracilis*

Tianyu Bai *et al.*

Corresponding author: Yumei Wang, wangym@iphy.ac.cn; Lirong Tian, tianlr@hebtu.edu.cn

Sci. Adv. **12**, eaea5561 (2026)
DOI: 10.1126/sciadv.aea5561

This PDF file includes:

Figs. S1 to S20
Tables S1 to S6

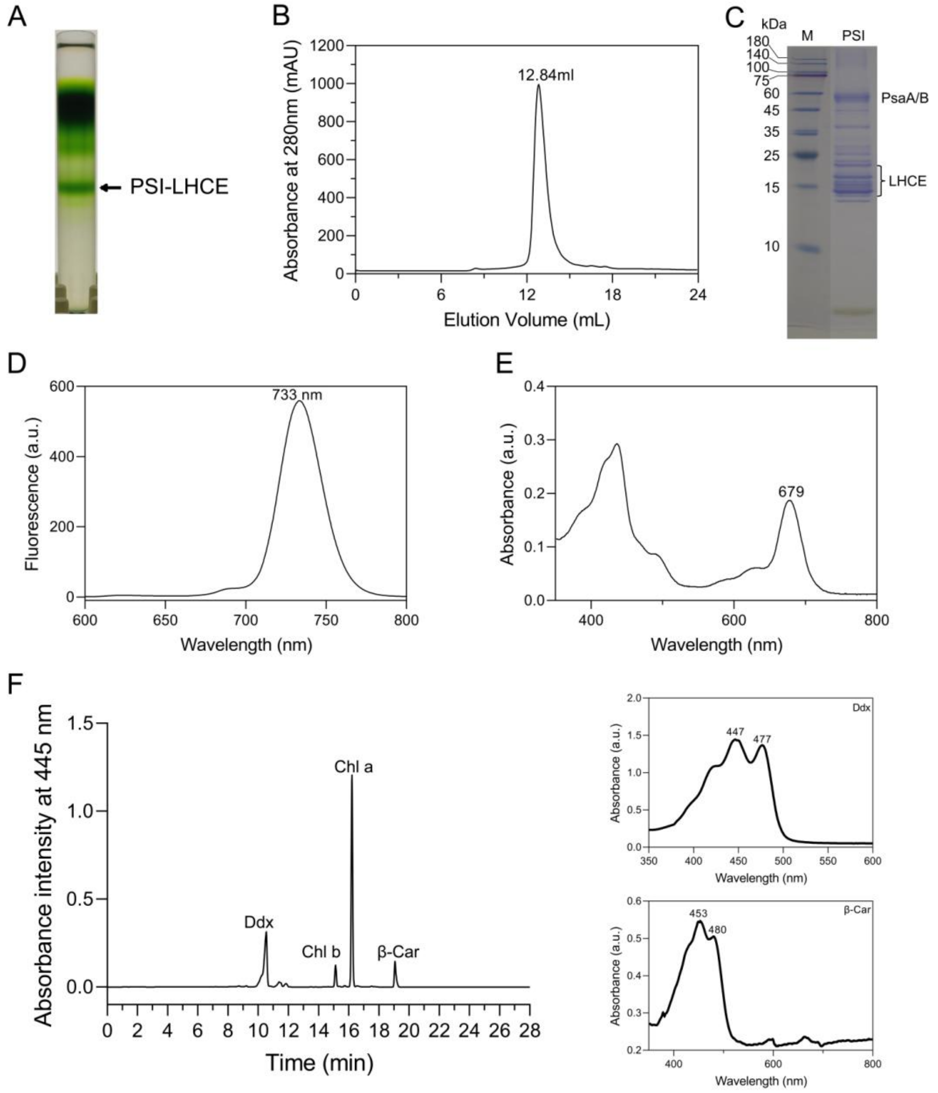

Fig. S1. Isolation and characterization of *E. gracilis* PSI-LHCE. (A) Isolation of the PSI-LHCE supercomplex by sucrose density gradient centrifugation (SDG). Samples corresponding to 0.5 mg Chls were loaded on top of each tube. **(B)** Size-exclusion chromatographic (SEC) elution profile of the PSI-LHCE fraction isolated by SDG from *E. gracilis*. Elution was performed at 4 °C with a Superose 6 Increase 10/300 GL column and monitored by absorption at 280 nm. **(C)** SDS-PAGE analysis of the PSI-LHCE supercomplex from *E. gracilis*. Lane 1, marker; lane 2, PSI-LHCE after SEC (5 μg of Chls). **(D)** 77 K fluorescence spectra (excited at 436 nm) of PSI-LHCE. **(E)** Absorption spectra of the PSI-LHCE supercomplex at room temperature. **(F)** HPLC analysis of pigments of the PSI-LHCE supercomplex from *E. gracilis*. Major pigment peaks were identified as Chl *a*, Chl *b*, diadinoxanthin (Ddx), and β-carotene (β-Car), based on their elution time and characteristic absorption spectra. The data are based on three independent experiments, each producing similar results.

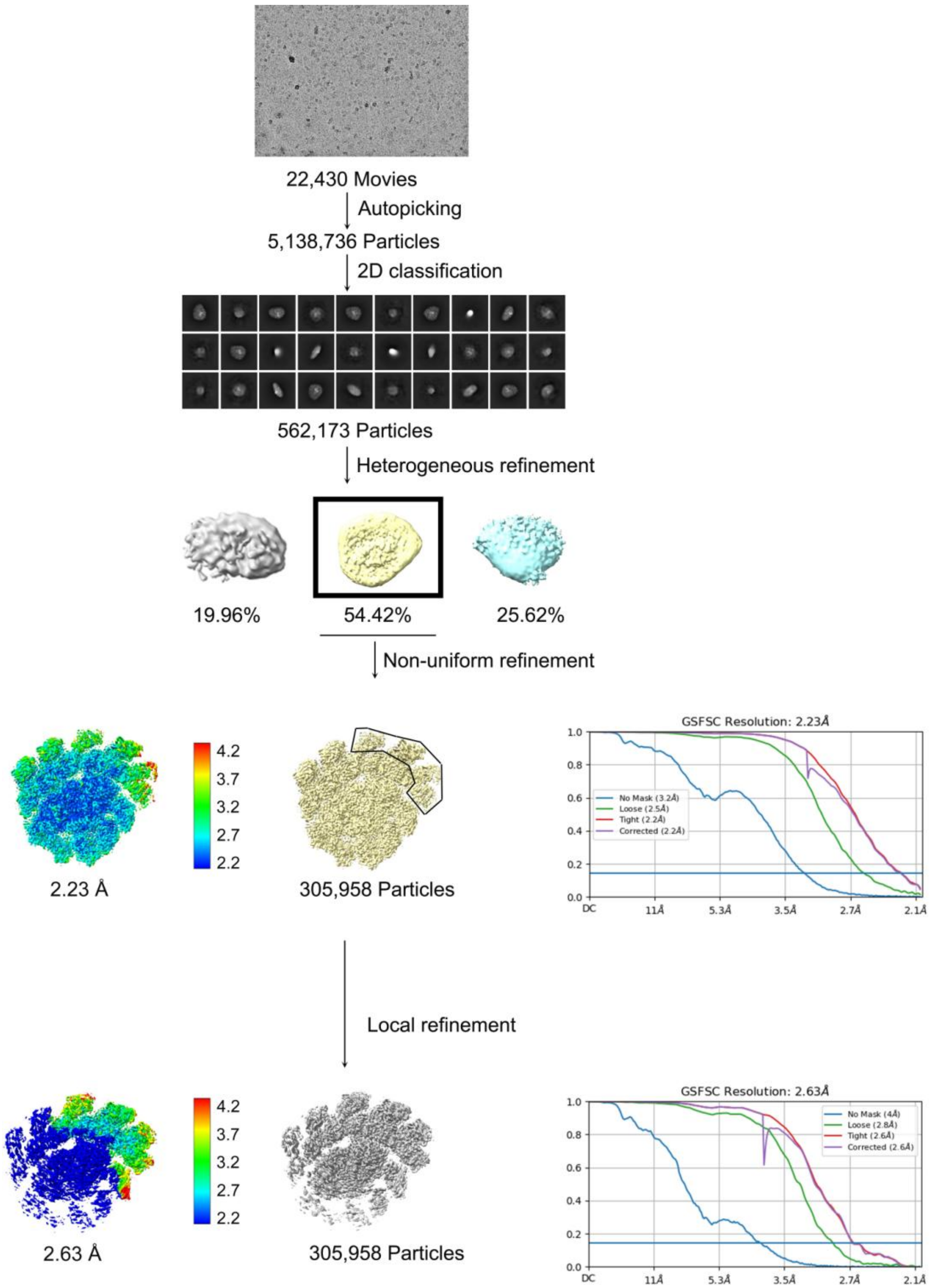

Fig. S2. Cryo-EM data collection, processing, refinement, and validation of the *E. gracilis* PSI-LHCE supercomplex structure. Global and local resolution maps of the PSI-LHCE were generated using cryoSPARC. The relatively flexible LHCE subunits (LHCE 6/14/15/16) were further improved via local refinement. The maps are colored according to their local resolution (Å) as indicated by the color bars. The gold-standard FSC curves of the final 3D reconstruction are shown for the global and local maps, respectively.

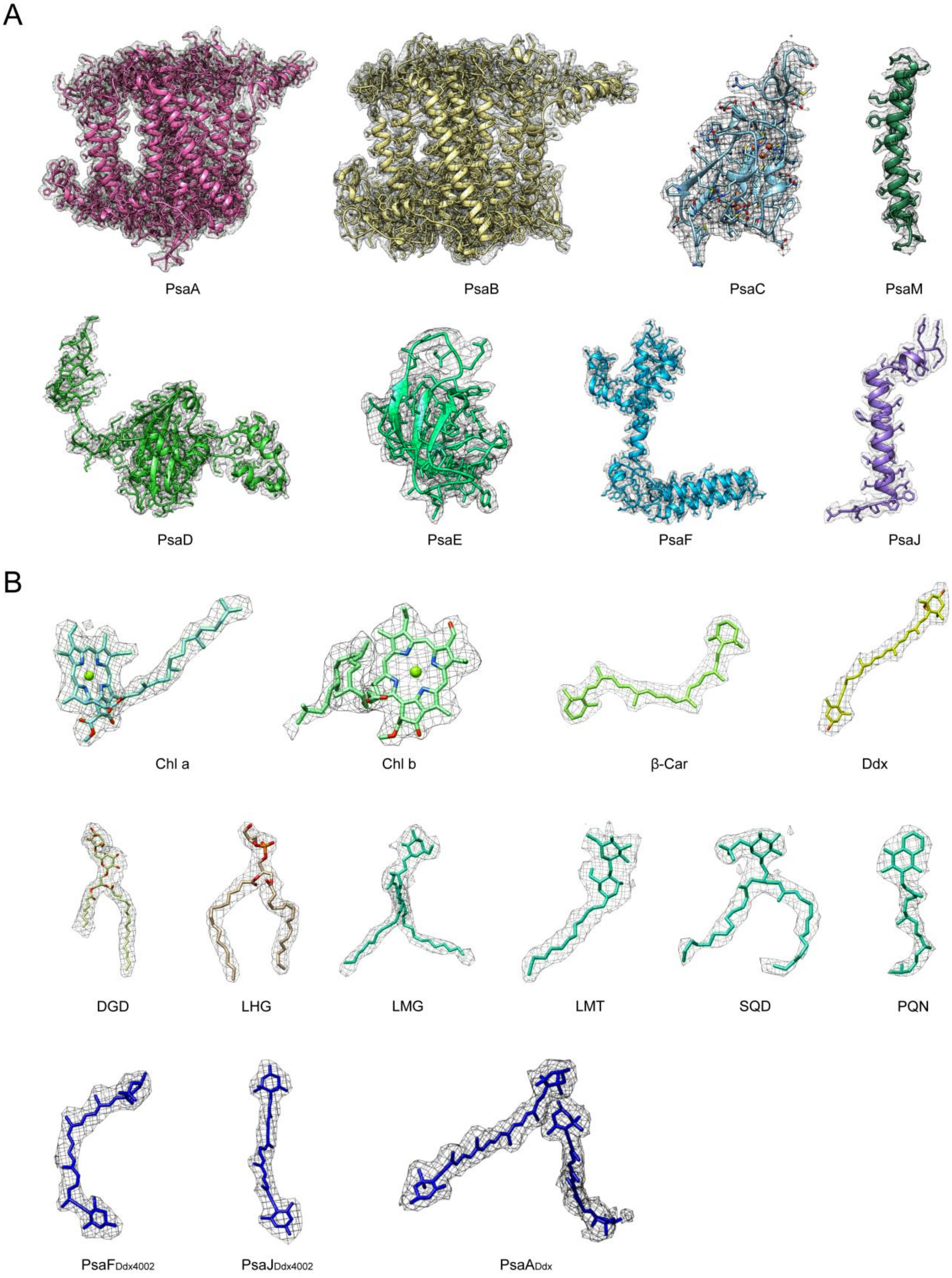
A
PsaA
PsaB
PsaC
PsaM
PsaD
PsaE
PsaF
PsaJ
B
Chl a
Chl b
β-Car
Ddx
DGD
LHG
LMG
LMT
SQD
PQN
PsaFDdx4002
PsaJDdx4002
PsaADdx

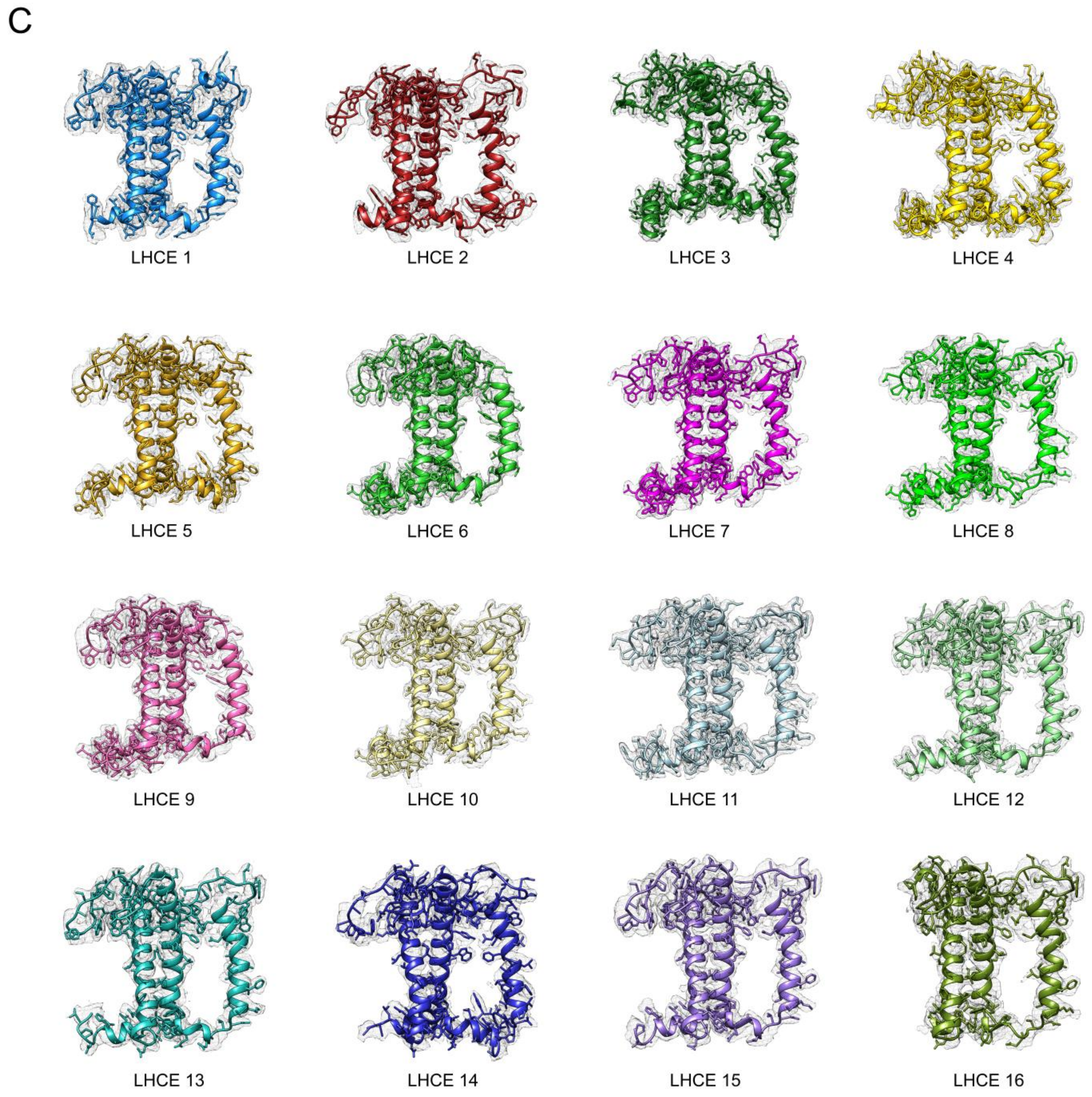

Fig. S3. Cryo-EM density maps of protein subunits and representative cofactors in the *E. gracilis* PSI-LHCE supercomplex. (A) Cryo-EM density maps and structures of core subunits. **(B)** Cryo-EM maps of pigments and lipids. **(C)** Cryo-EM density maps and structures of LHCE subunits. The PSI core and LHCE subunits are shown as ribbon models. Pigments, lipids, and other ligands are represented as stick models. β-Car, β-carotene; Ddx, diadinoxanthin; DGD, digalactosyl diacylglycerol; LHG, 1,2-dipalmitoyl-phosphatidyl-glycerol; LMG, 1,2-distearoyl-monogalactosyl-diglyceride; LMT, dodecyl-β-D-maltoside; SQD, sulfoquinovosyldiacylglycerol; PQN, phylloquinone.

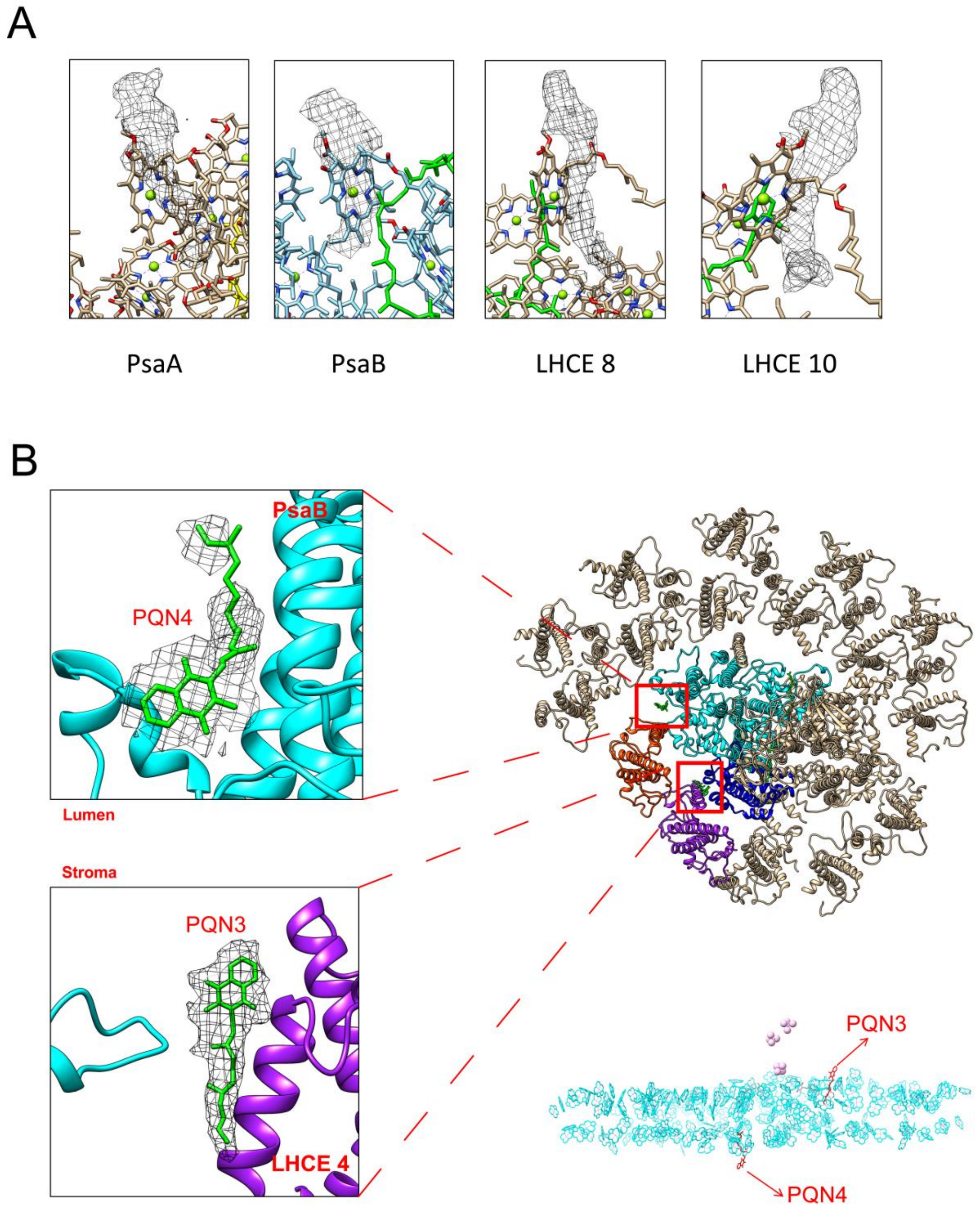

Fig. S4. Unassigned densities. (A) Some discrete densities were identified in the PSI core and LHCEs. **(B)** Two extra phylloquinone (PQN) molecules are shown with their corresponding cryo-EM densities. The head group of PQN3 faces the stromal side, whereas the head group of PQN4 is oriented towards the luminal side.

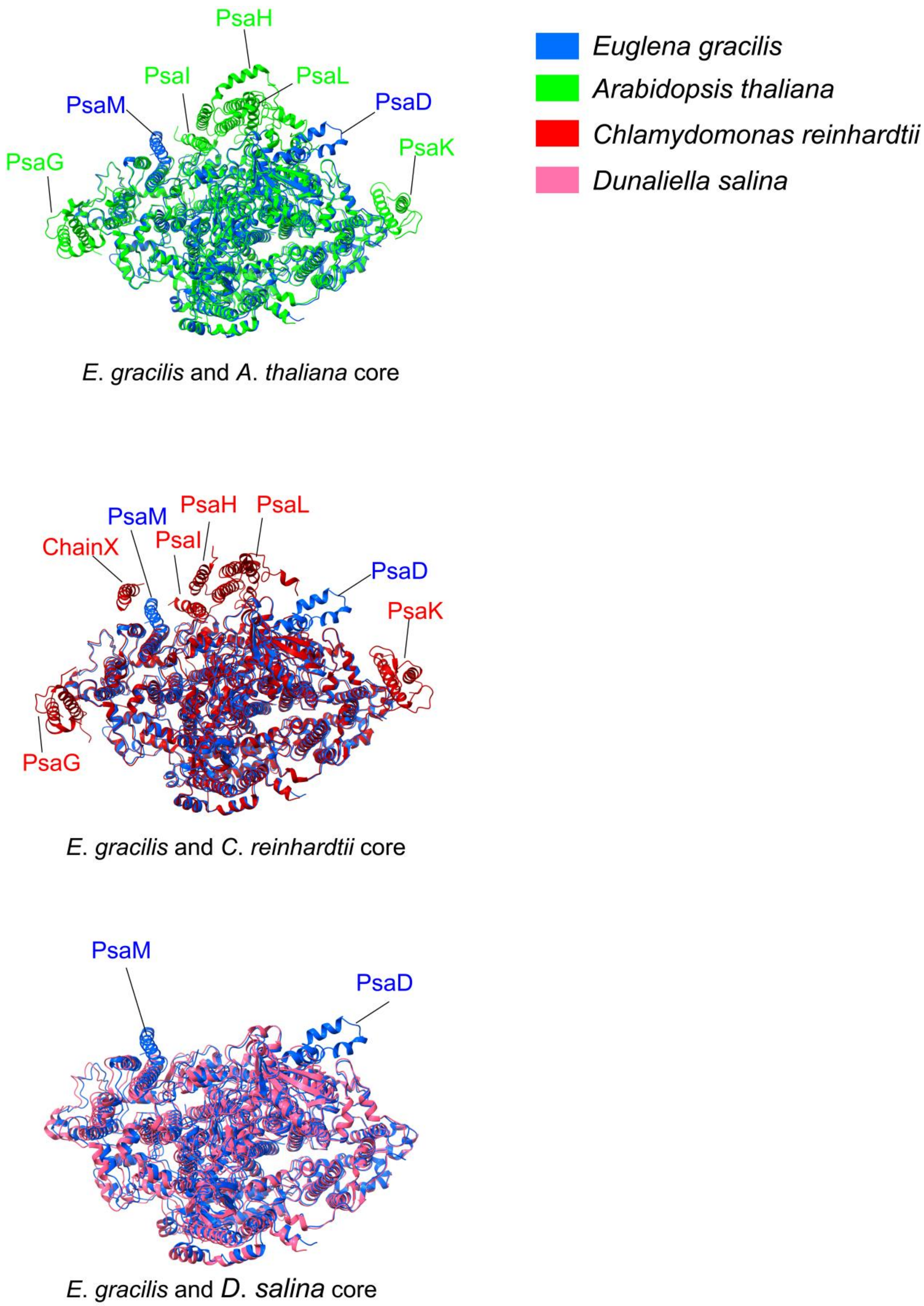

Fig. S5. Superposition of the *E. gracilis* core complex with those from *A. thaliana* (PDB: 7WFE), *C. reinhardtii* (PDB: 6IJO), and *D. salina* (PDB: 6YXR).

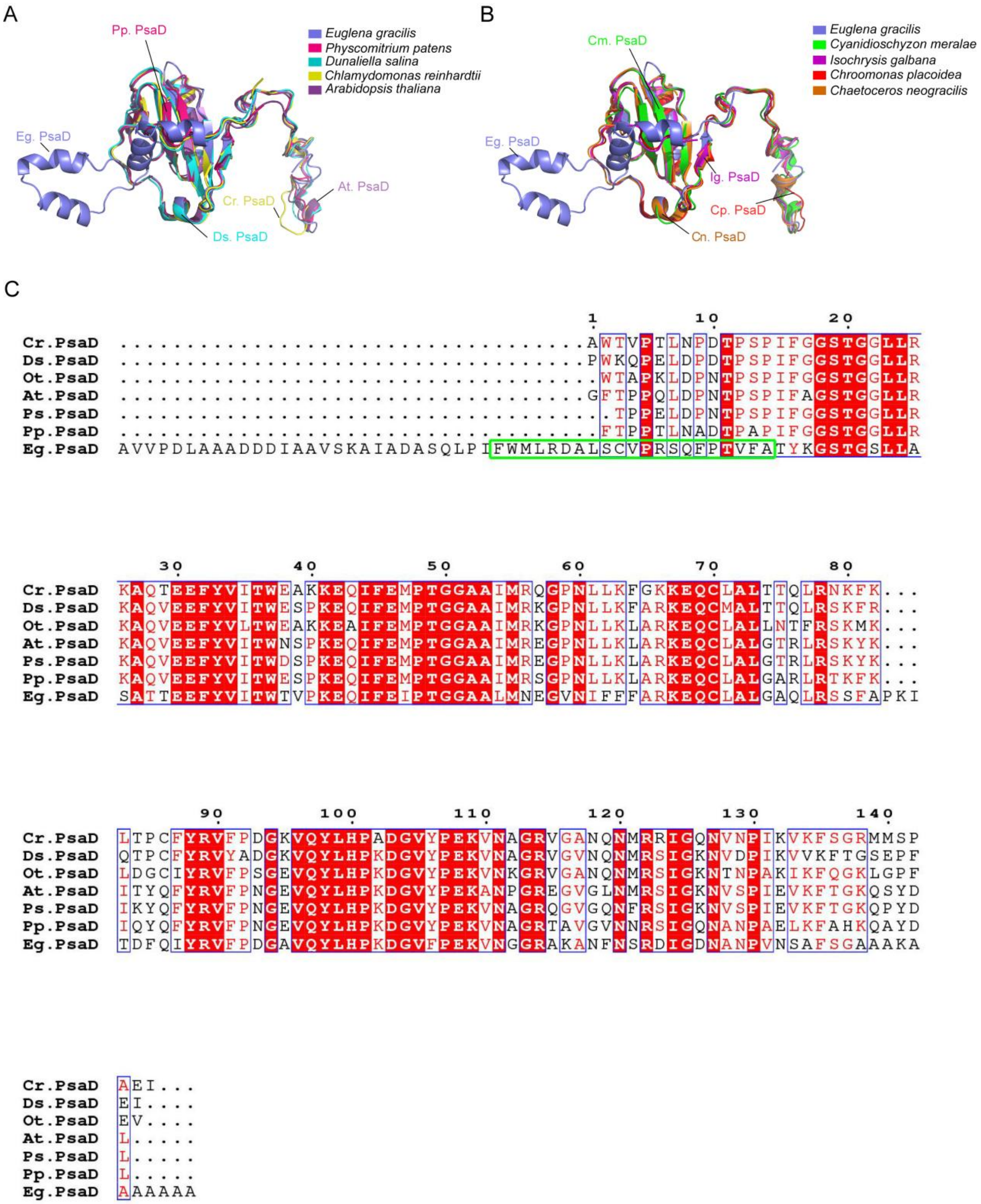
A
Pp. PsaD
Euglena gracilis
Physcomitrium patens
Dunaliella salina
Chlamydomonas reinhardtii
Arabidopsis thaliana
Eg. PsaD
Cr. PsaD
At. PsaD
Ds. PsaD
B
Cm. PsaD
Euglena gracilis
Cyanidioschyzon meralae
Isochrysis galbana
Chroomonas placoidea
Chaetoceros neogracilis
Eg. PsaD
Ig. PsaD
Cp. PsaD
Cn. PsaD
C
1 10 20
Cr.PsaD .....................................AWTVPTLNPDTPSPIFGGSTGGLLR
Ds.PsaD .....................................PWKQPELDPDTPSPIFGGSTGGLLR
Ot.PsaD ......................................WTAPKLDPNTPSPIFGGSTGGLLR
At.PsaD .....................................GFTPPQLDPNTPSPIFAGSTGGLLR
Ps.PsaD ......................................TPPELDPNTPSPIFGGSTGGLLR
Pp.PsaD ......................................FTPPTLNADTPAPIFGGSTGGLLR
Eg.PsaD AVVPDLAAADDDIAAVSKAIADASQLPIFWMLRDALSCVPRSQFFPTVFATYKGSTGSLLA
30 40 50 60 70 80
Cr.PsaD KAQTEEFYVITWEAKKEQIFEMPTGGAAIMRQGPNLLKFGKKEQCLALTTQLRNKFK...
Ds.PsaD KAQVEEFYVITWESPKEQIFEMPTGGAAIMRKGPNLLKFARKEQCMALTTQLRSKFR...
Ot.PsaD KAQVEEFYVLTWEAKKEAIFEMPTGGAAIMRKGPNLLKLARKEQCLALLNTFRSKMK...
At.PsaD KAQVEEFYVITWNSPKEQIFEMPTGGAAIMREGPNLLKLARKEQCLALGTRLRSKYK...
Ps.PsaD KAQVEEFYVITWDSPKEQIFEMPTGGAAIMREGPNLLKLARKEQCLALGTRLRSKYK...
Pp.PsaD KAQVEEFYVITWESPKEQIFEMPTGGAAIMRSGPNLLKLARKEQCLALGARLRTKFK...
Eg.PsaD SATTEEFYVITWTVPKEQIFEIPTGGAALMNEGVNIFFFARKEQCLALGAQLRSSFAPKI
90 100 110 120 130 140
Cr.PsaD LTPCFYRVFPDGKVQYLHPADGVYPEKVNAGRVGANQNMRRIGQNVNPIKVKFSGRMMSP
Ds.PsaD QTPCFYRVYADGKVQYLHPKDGVYPEKVNAGRVGVNQNMRSIGKNVDPIKVVKFTGSEPF
Ot.PsaD LDGCIYRVFPSGEVQYLHPKDGVYPEKVNKGRVGANQNMRSIGKNTNPAKIKFQGKLGPF
At.PsaD ITYQFYRVFPNGEVQYLHPKDGVYPEKANPGREGVGLNMRSIGKNVSPIEVKFTGKQSYD
Ps.PsaD IKYQFYRVFPNGEVQYLHPKDGVYPEKVNAGRQGVGQNFRSIGKNVSPIEVKFTGKQPYD
Pp.PsaD IQYQFYRVFPNGEVQYLHPKDGVYPEKVNAGRTAVGVNNRSIGQNANPAELKFAHKQAYD
Eg.PsaD TDFQIYRVFPDGAVQYLHPKDGVFPEKVNGGRAKANFNSRDIGDNANPVNSAFSGAAAKA
Cr.PsaD AEI...
Ds.PsaD EI....
Ot.PsaD EV....
At.PsaD L.....
Ps.PsaD L.....
Pp.PsaD L.....
Eg.PsaD AAAAAA

D

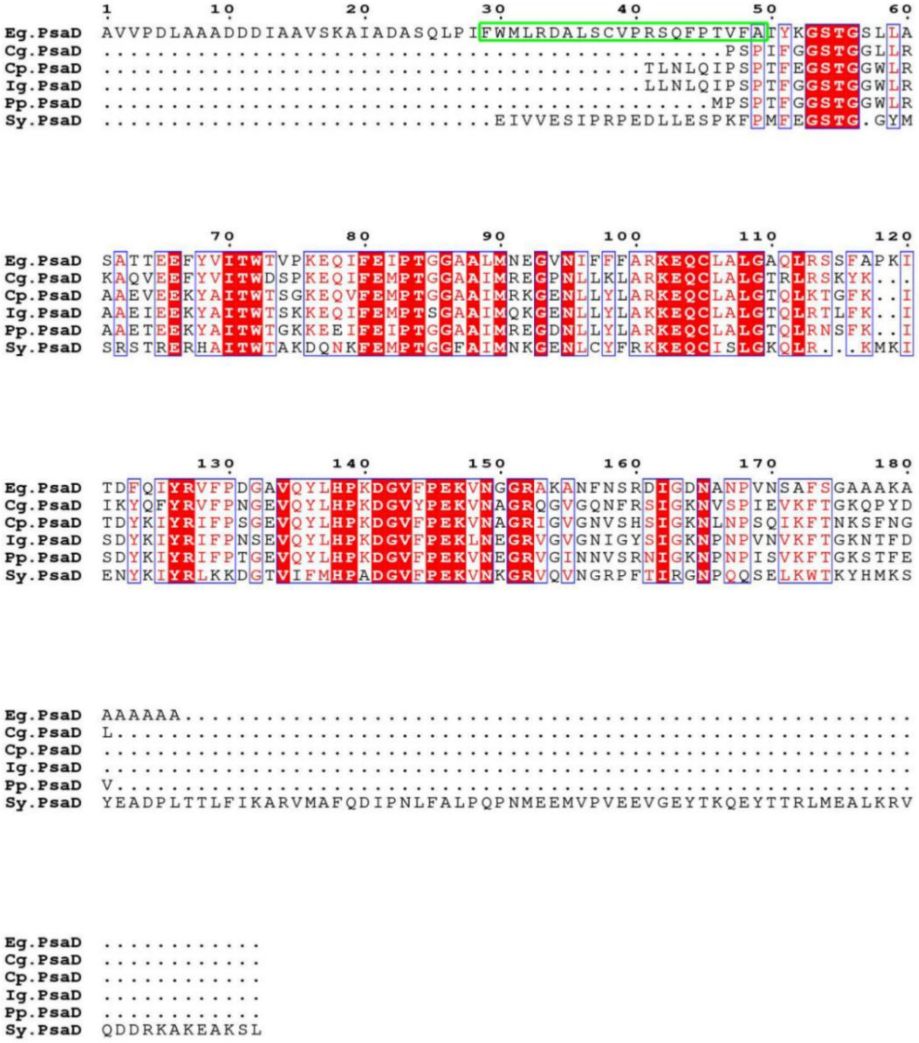

Fig. S6. Comparison of PsaD from *E. gracilis* with homologs from green algae, mosses, higher plants, haptophytes, red algae, cryptophytes, diatoms, and dinoflagellates. (A) Superposition of *E. gracilis* PsaD with PsaD from *D. salina* (PDB: 6YXR), *C. reinhardtii* (PDB: 6IJO), *Physcomitrium patens* (PDB: 7KSQ), and *A. thaliana* (PDB: 7WFE). **(B)** Superposition of *E. gracilis* PsaD (purple) with PsaD from *Cyanidioschyzon merolae* (PDB: 5ZGB), *Chroomonas placoidea* (PDB: 7Y7B), *Chaetoceros gracilis* (PDB: 6LY5), and *Isochrysis galbana* (PDB: 8Z11). **(C)** Sequence alignment of PsaD from *E. gracilis* (Eg), *D. salina* (Ds), *O. tauri* (Ot), *A. thaliana* (At), *Pisum sativum* (Ps), and *C. reinhardtii* (Cr). **(D)** Sequence alignment of PsaD from *E. gracilis* (Eg), *Symbiodinium* sp. (Sy), *Isochrysis galbana* (Ig), *Porphyridium purpureum* (Pp), *Chroomonas placoidea* (Cp), and *Chaetoceros gracilis* (Cg). The green box highlights the two additional N-terminal α-helices unique to the *Eg* PsaD subunit.

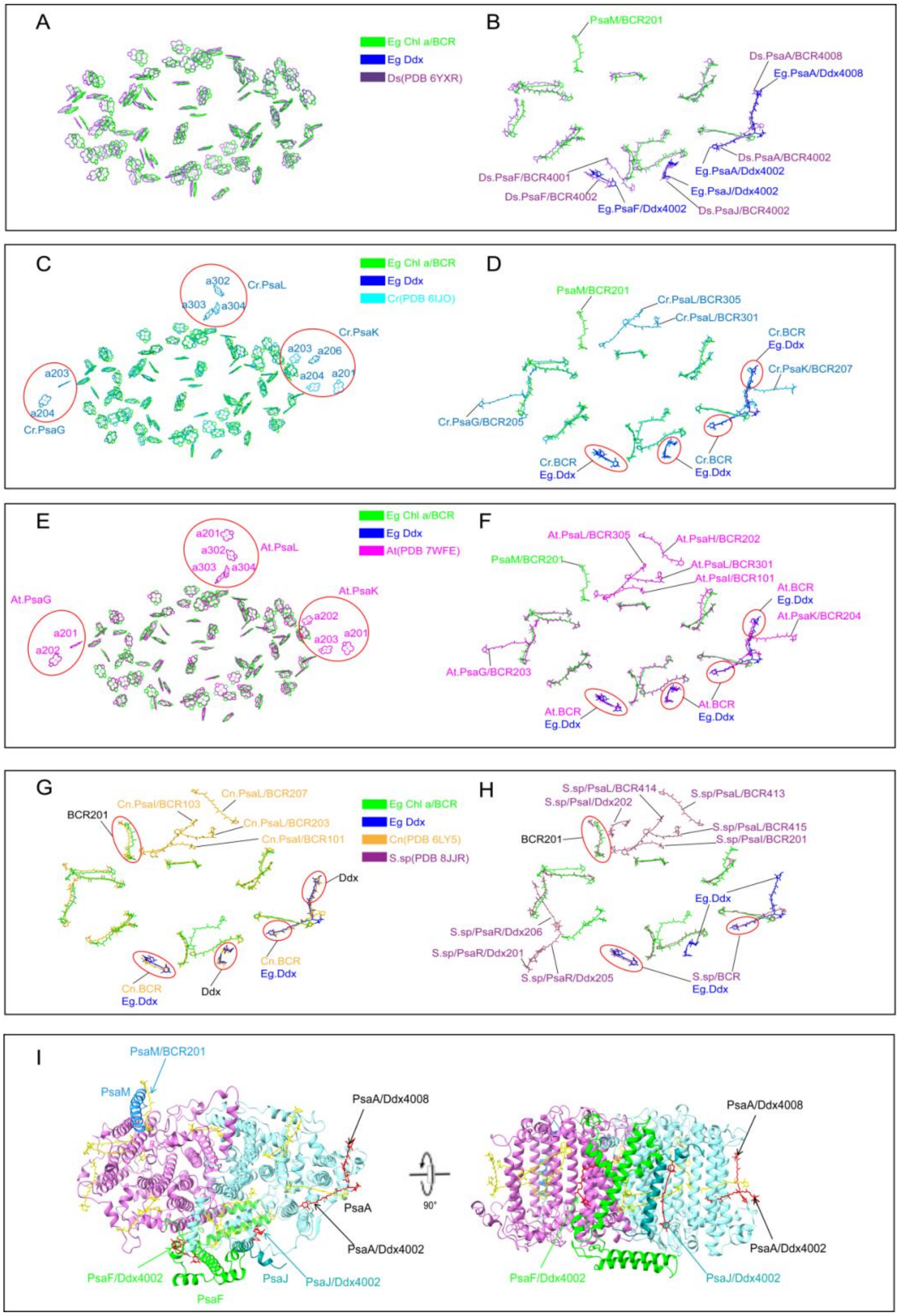

Fig. S7. Comparison of pigment arrangements in the *E. gracilis* PSI core with those in green- and red-lineage photosynthetic organisms. (**A to F**) Superposition of Chl and carotenoid sites in the *E. gracilis* PSI core with those in *D. salina* **(A and B)**, *C. reinhardtii* **(C and D)**, and *A. thaliana* **(E and F)**. (**G and H**) Carotenoid sites in diatoms **(G)** and dinoflagellates **(H)** (PDB: 8JJR). (**I**) The binding sites of Ddx and PsaM/β-Car in the *E. gracilis* PSI core. All panels are viewed from the stromal side.

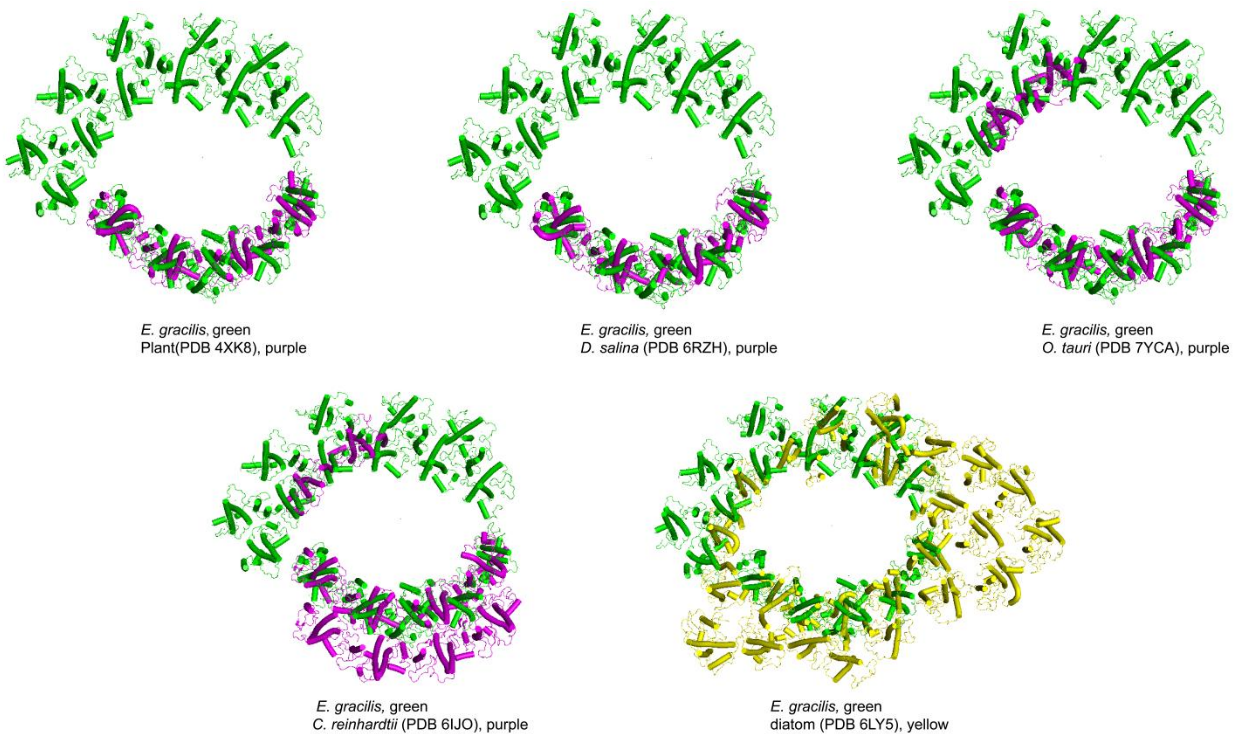

Fig. S8. Superposition of the *E. gracilis* LHCE with LHCIs from green algae (*D. salina*, *O. tauri*, and *C. reinhardtii*), higher plants, and diatoms.

Fig. S9. Protein sequences of the PSI core and LHCE subunits in the *E. gracilis* PSI-LHCE supercomplex structure (PDB: 9V7T and 9V7U). Sequences of PsaA, PsaB, PsaC, PsaM, PsaJ, LHCE 1–4, and LHCE 6–16 were derived from *E. gracilis*. Due to the lack of available sequence data for *E. gracilis*, PsaD, PsaE, and PsaF were modeled using sequences from *C. reinhardtii* (PDB: 6IJO).

PsaA (Uniprot：P19430)

KRVRVAFVSNPVATSFEKWSRPGHFSRLLSKGPNTTTWIWNLHADAHDFDNHTTDLEDI
SRKVFSAHFGQLAIIEIWLSGMFFHGARFSNYEAWLLDPIHVKPSAQIVWPIVGQEILNG
DVGGNFQGIQITSGLFQLWRSCGITSEFQLYITALTGLIFSAVLFFAGWFHYHKAAPKLE
WFQNVESMLNHHLSGLLGLGCLSWAGHQIHVSLPINKLLDSGVNPAELPLPHDFILDKS
LISQLYPSFSKGLAPFFTFHWSEYSDFLTFRGGLNNVTGGLWLTDVAHHHLALAVLFILA
GHMYKTNWKIGHDIKGLLESHTGPFTGQGHKGLYEIFTNSWHAQLSLNLAMMGSLSIIV
AQHMYSMPPYPYIAIDYGTELSLFTHHYWIGGFCIVGAAAHAAIFMVRDYDPALNFNNL
LDRVLLHRDAIISHLNWVCIFLGLHSFGLYIHNDTLSALGRPQDMFSDSAIQLQPVFAQW
IQNTHYLAPTLTAFNLVSPTTPVWGGDVVSISGKVAMMPIKLGTADFLVHHIHAFTIHVT
VLILLKGVLFSRSSRLIPDKASLGFRFPCDGPGRGGTCQVSAWDHVFLGLFWMYNSISVA
IFHFSWKMQSDVWGTVLANKVSHITGGNFSQGSLTINGWLRDFLWAQSSQVIQSYGSPL
SAYGLMFLGAHFVWAFTLMFLFSGRGYWQELIESIVWAHNKLKVAPNIQPRALSITQGR
AVGVAHYLLGGIATTWSFFLARIISVG

PsaB (Uniprot：P19431)

KFPKFSQGLAQDPTTRRIWFGIATSHDFESHDGMTENNLYQKIFASHFGQLAIIFLWTSG
NLFHVAWQGNFEQWIKDPLHIRPIAHAISDPHFGQPAIEAFTRSQFPGPVNIAYSGVYQW
WYTIGLRTNVDLYNGSMFLLFIATLALFAGWLHLEPKYSPKVSWFKDAESRLNHHLSAL
FGLSSLAWSGHLIHVAIPESRGIHVRWDNFLSQLPHPSGLEPFFKGNWSLYSENPDSLTH
VFGTASGAGTAVLTFLGGFHPETKSLWLTDIAHHHLAIAVLFIVAGHMYRTNFAIGHRID
DILNAHKAPSGKLGLGHFGLYETINNSLHFQLGLALASLGVITSLVAQHMYSLSPYAFLI
QDRTTMAALYTHHQYIAGFIMTGAFAHGAIFFIRDFDEEKNKGNVLSRILDHKEAIISHLS
WVTLFLGFHTLGLYVHNDVMQAFGTPEKQILIEPVFAQWIQSAHGKNIYELNILLSSDSS
NAFSASQAIWLPGWLNGINDKSTSLFLQIGPGDFLVHHAIALGLHTTTLILVKGALDARG
SRLMPDKKDFGYSFPCDGPGRGGTCDISAWDAFYLAVFWMLNTIGWTTFYWHWKHIT
LWQGNVGQFNESSTYLMGWLRDYLWLNSSQLINGYNPFGMNSLAVWGWMFLFGHLV
WATGFMFLISWRGYWQELIETLVWAHERTPITNVFKWTDKPVALSIVQARLVGLAHFS
VGYVFTYAAFLIASTSAKFG

PsaC (Uniprot：A0A0G3VS95)

SHSVKIYNTCIGCTQCVRACPTDVLEMVPWDGCKAGQIASSPRTEDCVGCKRCESACPT
DFLSVRVYLGSETSRSMGLAY

PsaD

AVVPDLAAADDDIAAVSKAIADASQLPIFWMLRDALSCVPRSQFPTVFATYKGSTGSLL
ASATTEEFYVITWTVPKEQIFEIPTGGAALMNEGVNIFFFARKEQCLALGAQLRSSFAPKI
TDFQIYRVFPDGAVQYLHPKDGVFPEKVNGGRAKANFNSRDIGDNANPVNSAFSGAAA
KAAAAAAA

PsaE

AGPALGSVVKILRPESYWYNQFGTVIAVDASGGIRYSVVVRFAKQNYEGVTVNNFALD
ELVAA

PsaF

AEAILGLTPCSDSAMFQEVLVKEVKALNQRENLYDAGSAPYLALKATKAKTQARFANY
AVAGLLCGADGLPHLIADPGLAVKYGHAGEVDIPTIGFLYVAGYIGVAGRVYLQTISTR
DNPTEKEIIINVPLATSIAFKSWDWPDEAVQELAAGTLLESAMNVPTAKGA

PsaM (Uniprot：P31479)

MEITTNQVVYIALLASLIPAFFAFKLGKSLNQ

PsaJ (Uniprot：P30394)

MKYFTTYLSTAPVVAVLWFTLTASLLIEINRFFPDIL

LHCE 1 (Uniprot: A8HPE8, Lhca11)

RATWFPGAKAPPHLTGEFPGDRGFDPVDFAANPESFERMRASEVFHGRMAMLGVAGCL
IPELLGRGVWFQAGESVDAAQLGLYFMFLAAPSEYWRGNGGFGWDQKEKKAGNRIYP
GFDPLKMTSDETKTKEIKNGRLAMIAFLGLVSQANTTGVSPFQNLAAVFGA

LHCE 2 (Uniprot: A8HPE8, Lhca11)

WFPGATPPAYLTGEFPADRGFDPLGLAKDPATYERMRSSEVFHSRLAMLGVVGSLIPEL
QGQGAWYTLSEKQFGPGPNGEVIGFTELALIGMLFSYPLEYWRGNGGFGWFEEKGDRT
YPGFDPLQLTSEYTKTAEIKNGRLAMSALLGFAVQHATTGASPLENWAATTG

LHCE 3 (Uniprot: A4QPH9, Lhcbm3)

LSDWYGPNRKLFLGPLSDGAPEHLKGELPGDYGFDVLGLATQPTRLERYRQGEIINGRW
AMLGIVGCIVPELLARNYGVPFPEPVWFKTGATVFSEEGLNYLGNPSLIHAKSIAAILVTE
ILFVGAAEAFRVSGGPLGPATDLVYPGKAFDPLGLSKDATAFAELKVKEVKNGRLAML
GMLGLFMQGFATGKGPLQNLADHLADPTGANIITFHQRVLSDL

LHCE 4 (Uniprot: A4QPH8, Lhcbm1)

GSKWYGPNRPKWLGPLSGGAVPEYLKGEYAGDYGFDTAGLAADPKLFQRYRDAELQN
GRWAMLGVLGCLAPEVLSNVFGVPYPEPVWFKTGATILNGGSIDYLANPKLIHASNLLL
TLVLELVFFFAAETWREAGEGPLGKAQDKSYPGGVFDPLGLSKDPAAFAEAKVKEVKN
GRLAMLAMLGLFVQAGVTGQSPLENLSAHLANPGVNFWTSYAPTLA

LHCE 5 (Uniprot: A8HPH1, Lhcbm10)

RPTWFPGAEAPTWLTGEYPGDRGFDPFGLAKDPADFAKFRDSEVFHGRWAMLGLVGC
LVPEVFGNLGIAQLPAWYDAATVANTSNLDYLGNPNLVHASNVSFIALSTLLLMGPVEA
WRWNGALASEAKSAERTTYPGGPFDPLKLGASPELKLKEIKNGRLAMVGMFGFWAQS
YVTGEGPLANLAAHLADPAHNNLLNT

LHCE 6 (Uniprot: A8HPD3, Lhca7)

RPLWFPGGYAPSWLTGQHFGDRGFDPAGLAADPKVFERMRVSEVYHGRLAMLAIVGA
VVPDIQGKGAWYEAAQSAGIGVNEVAVFTAAYGIAEVARGLKANSDPTSRYSGFDSLN
LTTDYTKEAEIKNGRLALTAMLGLQVQRHVTGVSPLLNLVEHVKHPLVHTIAESVMHQ
W

LHCE 7 (Uniprot: A8HPD0, Lhca6)

RGLWFPNITPPAYLTGEFPADRGFDPAGLAADPKVYERMRIAEVFNGRLAMLAIVGCVY
PELLGNGVWFEVWNKVDFYRFALISLQVVAPLEYWRGNGGFGWDGEEKYDRSYPGFD
PCNLTTEYTKAAEIKNGRLAMIGMFGLEVQSHVTAQGPVANLIEHLRHPLAANIGANLA
HPW

LHCE 8 (Uniprot: A8HPD3, Lhca7)

RLLWIPNATPPAHLTGEFPGDRGFDPLGLAKDPKVYQRMRISEVFHGRLAMLGIVGCVA
PELFFSKGAWFDYSDYDLNRLGLIALQVIAPLEYWRGNGGFSWDGNDGPDRSYPGFDP
LGLTNEETKLQEIKNGRLAMTAMLGLEVQSHITGKSPLTNLSEHLSHPLSANLLTGG

LHCE 9 (Uniprot: A8HPD3, Lhca7)

RPLWFPGGYAPSWLTGQHFGDRGFDPAGLAADPKVFERMRVSEVYHGRLAMLAIVGA
VVPDIQGKGAWYEAAQSAGIGVNEVAVFTAAYGIAEVARGLKANSDPTKRYSGFDSLN
LTTDYTKEAEIKNGRLALTAMLGLQVQRHVTGVSPLVNLVEHVKHPLNHNIAESVMHQ
WPVA

LHCE 10 (Uniprot: A8HPD0, Lhca6)

EKYRGLWFPNITPPAYLTGEFPADRGFDPAGLAADPKVYERMRIAEVFNGRLAMLAIVG
CVYPELLGNGVWFEVWNKVDFYRFALISLQVVAPLEYWRGNGGFGWDGEEKYDRSYP
GFDPCNLTTEYTKAAEIKNGRLAMIGMFGLEVQSHVTAQGPVANLIEHLRHPLAANIGA
NLAHPWPP

LHCE 11 (Uniprot: A8HPC6, Lhca5)

RLLWIPNATPPAHLTGEFPGDRGFDPLGLSKDPKVFARMRISEVFHGRLAMLGIVGCVG
QEWLFNKGAWFDYSDFDLPRLGLIALQVIAPLEYWRGNGGFSWNGNDGPDRSYPGFDP
LGLTTEDTKLREIKNGRLAMSAMLGLEVQSHITGKSPLTNLGDHLSSPFTANILT

LHCE 12 (Uniprot: A8HPD0, Lhca6)

HKDGVWFPGATPPAHLTGEYPADRGFDPLSLAADPTVYARMRVSEVFHARLSMLAIVG
AIVPEVLGKGAWFEAGNSVDGIKLGFIAMAIAAPTEYWRGNGGFNWDKGAADRSYPGF
DPLKLTTDYTKAAEIKNGRLAMAGLLGLTFQYLATGESPLANLAAHLANPV

LHCE 13 (Uniprot: A8HPD0, Lhca6)
HKDGVWFPGATPPAHLTGEYPADRGFDPLSLAADPTVYARMRVSEVFHARLSMLAIVG
AIVPEVLGKGAWFEAGNSVDGIKLGFIAMAIAAPTEYWRGNGGFNWDKGAADRSYPGF
DPLKLTTDYTKAAEIKNGRLAMAGLLGLTFQYLATGESPLANLAAHLANPVG
LHCE 14 (Uniprot: A8HPH1, Lhcbm10)
TWFPGAEAPAWLTGEYPGDRGFDPLGLAKDPEDFAKNRDSEVYHGRWAMLGLVGCLV
PEVFGNLGIAQLPAWYEAGAVANTGSLDYLGNPNLVHASNVPLIFFTTLLLFLPIEAWR
WNGQIAPEAKSAERTTYPGGPFDPLKLGASPELKLKEIKNGRLAMVGMFGFWAQSYVT
GEGPLANLAA
LHCE 15 (Uniprot: A8HPD0, Lhca6)
DGVWFPGATPPAHLTGEYPADRGFDPLSLAADPTVYARMRVSEVFHARLSMLAIVGAI
VPEVLGKGAWFEAGNSVDGIKLGFIAMAIAAPTEYWRGNGGFNWDKGAADRSYPGFD
PLKLTTDYTKAAEIKNGRLAMAGLLGLTFQYLATGESPLANLAAHLANP
LHCE 16 (Uniprot: A8HPC6, Lhca5)
PPAHLTGEYPADRGFDPLSLAADPTVYARMRVSEVFHARLSMLAIVGSIVPELLGKGAW
FEVGNSVDGIKLGFILMAIAAPTEYWRGNGGFNWDKGTADRSYPGFDPLKLTTDYTKA
AEIKNGRLAMTGLLGLTFQYLATGESPLANL

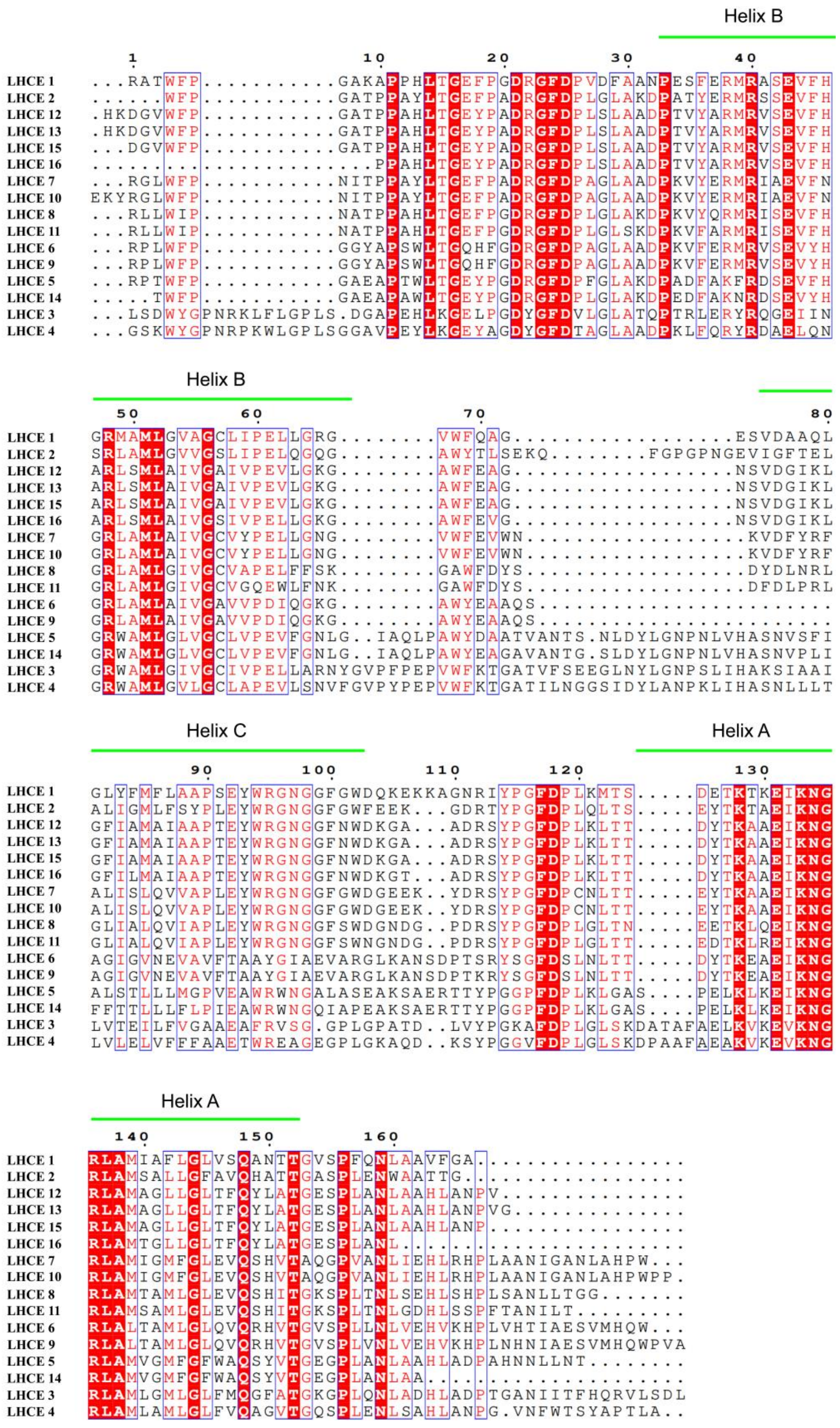

Fig. S10. Sequence alignment of LHCEs from *E. gracilis*. Secondary structure elements are indicated above the sequences. Fully conserved residues are shaded in red, and similar amino acids are highlighted by blue frames.

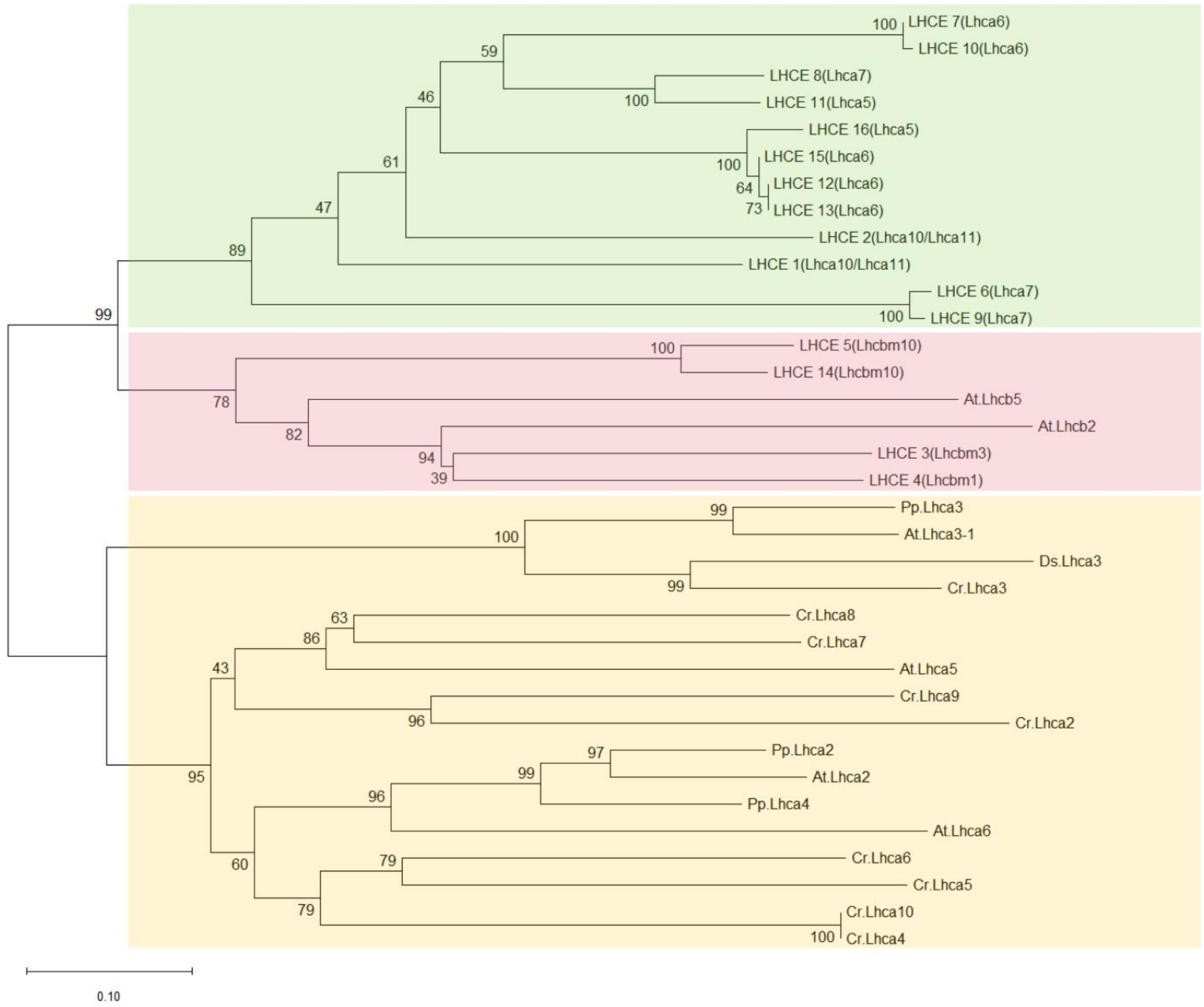

Fig. S11. Phylogenetic tree of LHCEs from *E. gracilis* (Eg) and LHCIs from *A. thaliana* (At), moss *P. patens* (Pp) , green alga *C. reinhardtii* (Cr) , and *D. salina* (Ds). The neighbor-joining (NJ) tree was constructed based on the amino acid sequences of LHCEs and LHCIs. The tree was built using the Poisson model, and a bootstrap test (1,000 replicates) was conducted to evaluate node support.

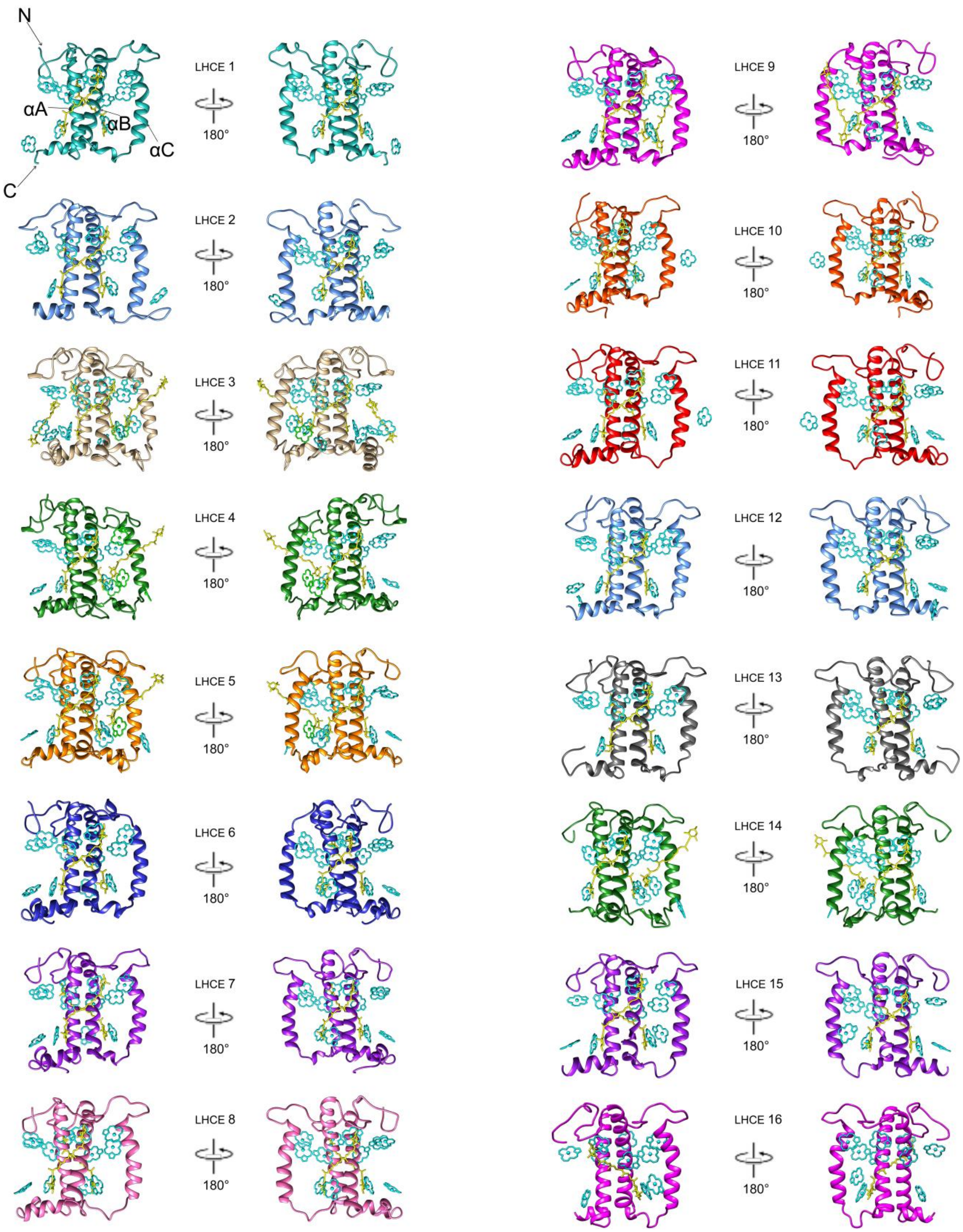

Fig. S12. Structures of individual LHCE subunits. Chl and Ddx molecules are colored cyan and yellow, respectively. The phytyl chains of Chls are omitted for clarity.

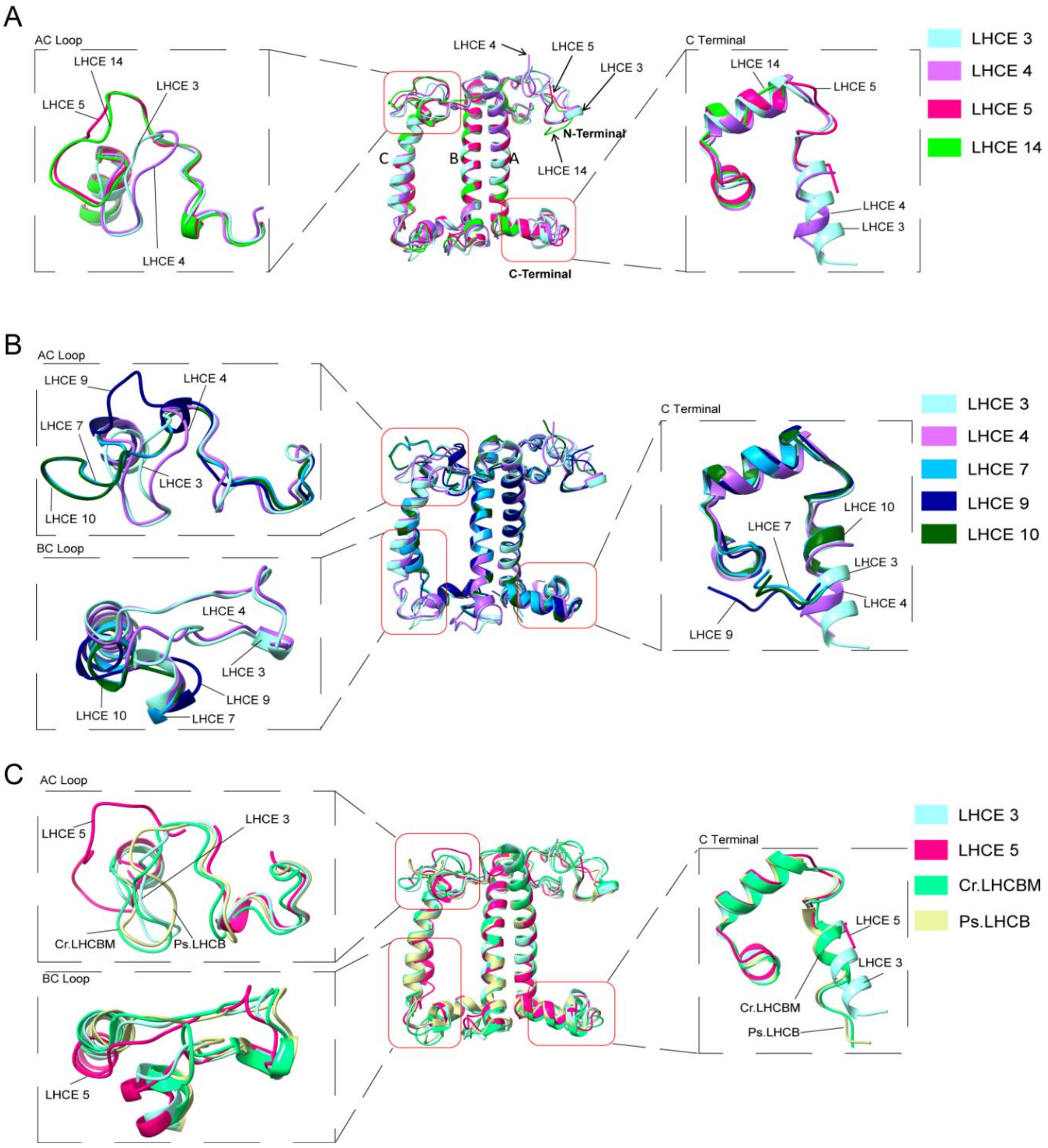
A
AC Loop
LHCE 14
LHCE 3
LHCE 5
LHCE 4
LHCE 4
LHCE 5
LHCE 3
N-Terminal
LHCE 14
C
B
A
C-Terminal
C Terminal
LHCE 14
LHCE 5
LHCE 4
LHCE 3
LHCE 3
LHCE 4
LHCE 5
LHCE 14
B
AC Loop
LHCE 9
LHCE 4
LHCE 7
LHCE 3
LHCE 10
BC Loop
LHCE 4
LHCE 3
LHCE 9
LHCE 10
LHCE 7
C Terminal
LHCE 10
LHCE 7
LHCE 3
LHCE 4
LHCE 9
LHCE 3
LHCE 4
LHCE 7
LHCE 9
LHCE 10
C
AC Loop
LHCE 5
LHCE 3
Cr.LHCBM
Ps.LHCB
BC Loop
LHCE 5
C Terminal
LHCE 5
LHCE 3
Cr.LHCBM
Ps.LHCB
LHCE 3
LHCE 5
Cr.LHCBM
Ps.LHCB

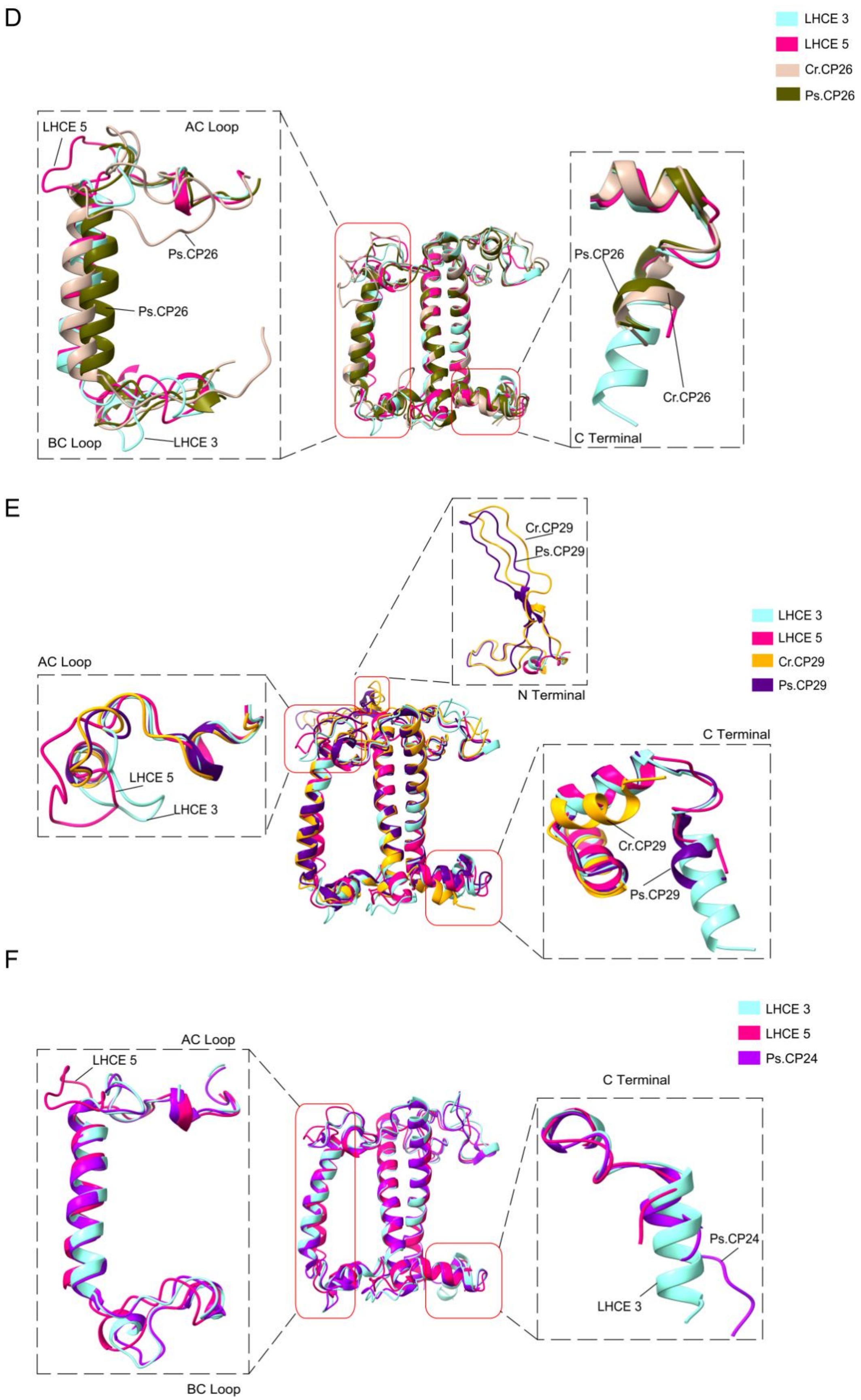
D
LHCE 3
LHCE 5
Cr.CP26
Ps.CP26
LHCE 5
AC Loop
Ps.CP26
Ps.CP26
BC Loop
LHCE 3
Ps.CP26
Cr.CP26
C Terminal
E
Cr.CP29
Ps.CP29
N Terminal
LHCE 3
LHCE 5
Cr.CP29
Ps.CP29
AC Loop
LHCE 5
LHCE 3
C Terminal
Cr.CP29
Ps.CP29
F
LHCE 3
LHCE 5
Ps.CP24
AC Loop
LHCE 5
BC Loop
C Terminal
Ps.CP24
LHCE 3

Fig. S13. Structural comparison of LHCE subunits with each other and with LHCIIs from green algae and higher plants. **(A)** Superposition of Lhcbm proteins from *E. gracilis*. **(B)** Superposition of Lhca (LHCE 7/9/10) and Lhcbm proteins (LHCE 3/4) from *E. gracilis*. **(C)** Comparisons of LHCE 3/5 with the major LHCIIs from green algae (PDB: 6KAF) and higher plant (PDB: 5XNL). **(D-F)** Comparisons LHCE 3/5 with CP26, CP29, and CP24 from green algae and higher plant.

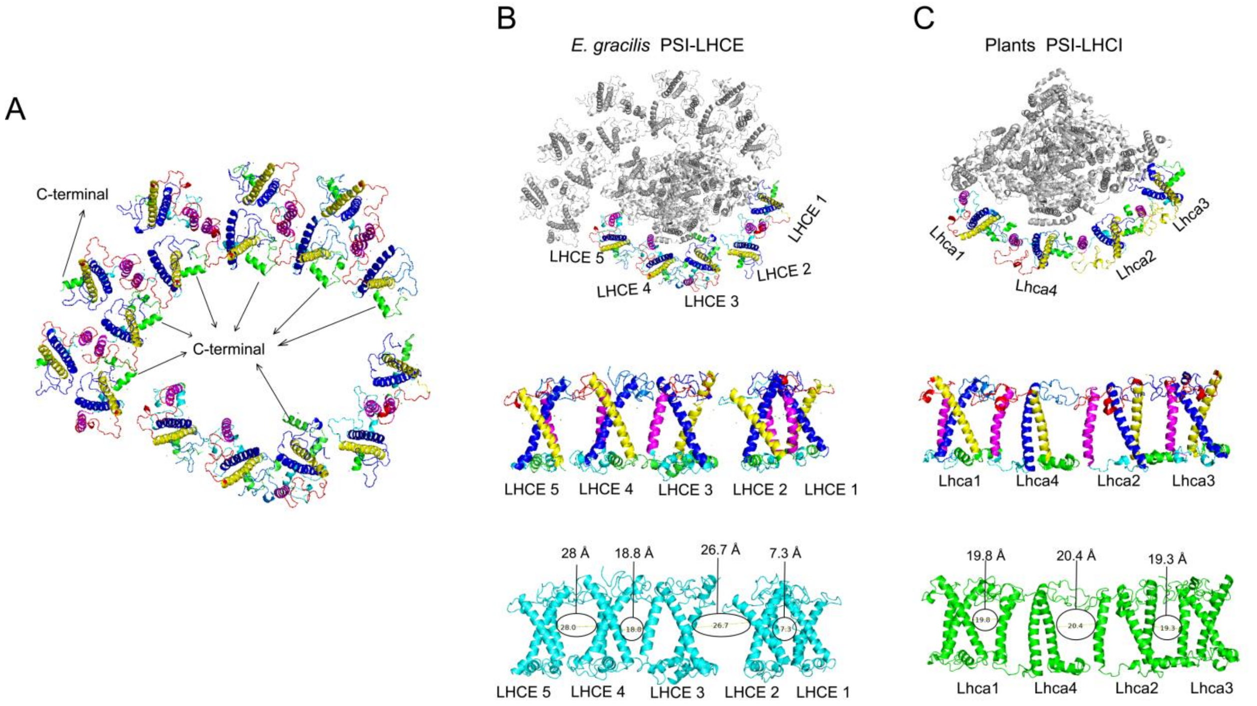

Fig. S14. Comparison of the spatial orientations of LHCEs in *E. gracilis* and LHCIs in higher plants (PDB: 4XK8). Helix B, C and A in plant LHCI and *E. gracilis* LHCE is colored by blue, magenta and yellow, respectively. The C-terminal helix is colored by green.

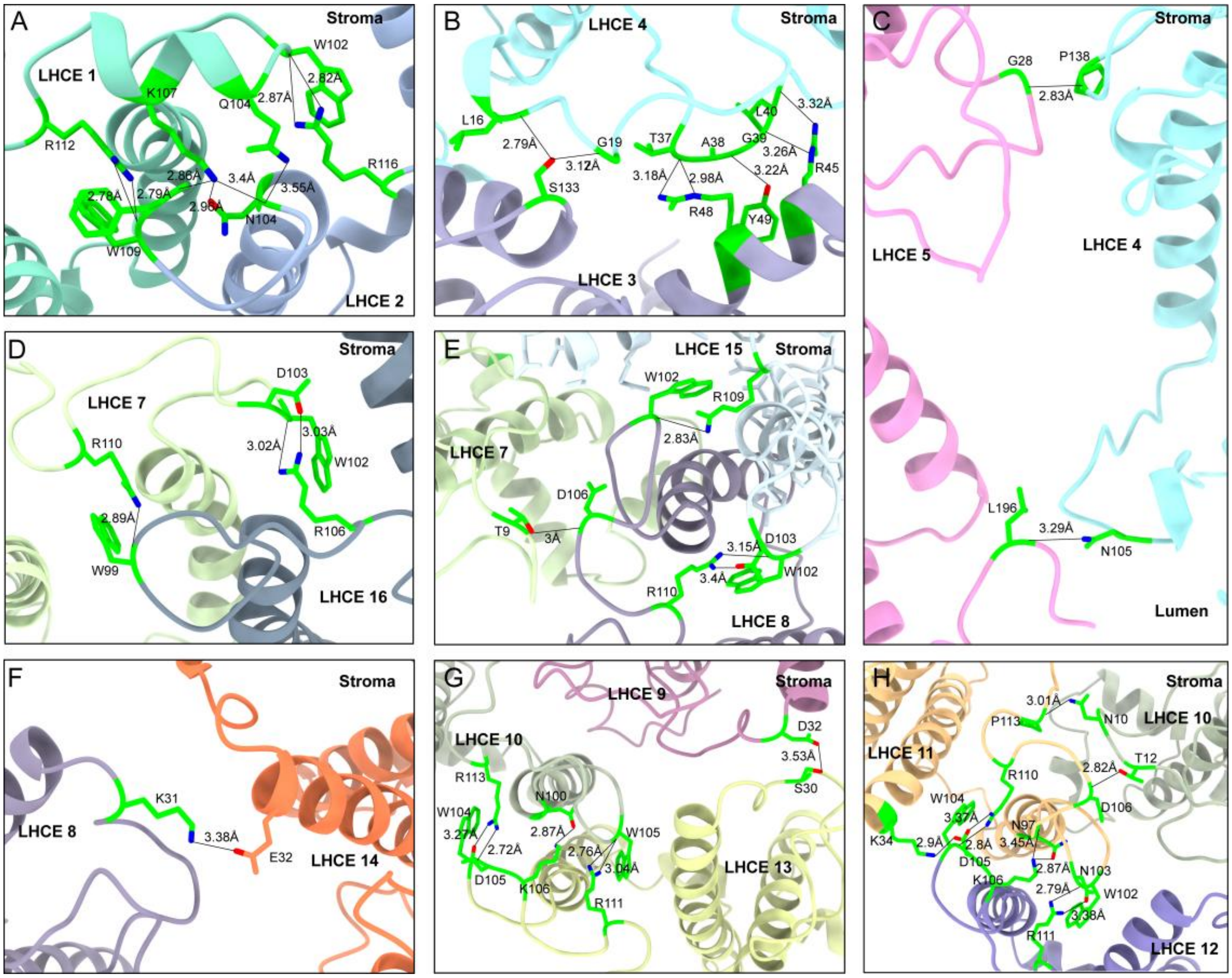

Fig. S15. Interactions among LHCE subunits in the PSI-LHCE. (A) Interactions between LHCE 1 and LHCE 2 at the stromal side. **(B)** Interactions between LHCE 3 and LHCE 4 at the stromal side. **(C)** Interactions between LHCE 4 and LHCE 5 at the stromal and luminal sides. **(D)** Interactions of LHCE 7 and LHCE 16 at the stromal side. **(E)** Interactions between LHCE 7 with LHCE 8 and LHCE 15 at the stromal side. **(F)** Interactions between LHCE 8 and LHCE 14 at stromal side. **(G)** Interactions between LHCE 13 with LHCE 9 and LHCE 10 at stromal side. **(H)** Interactions between LHCE 10 with LHCE 11 and LHCE 12 at stromal side. Interacting amino acid residues are depicted as green sticks; oxygen (O) and nitrogen (N) atoms are colored red and blue, respectively. Hydrogen bonds are indicated by solid black lines with distances labeled in Å.

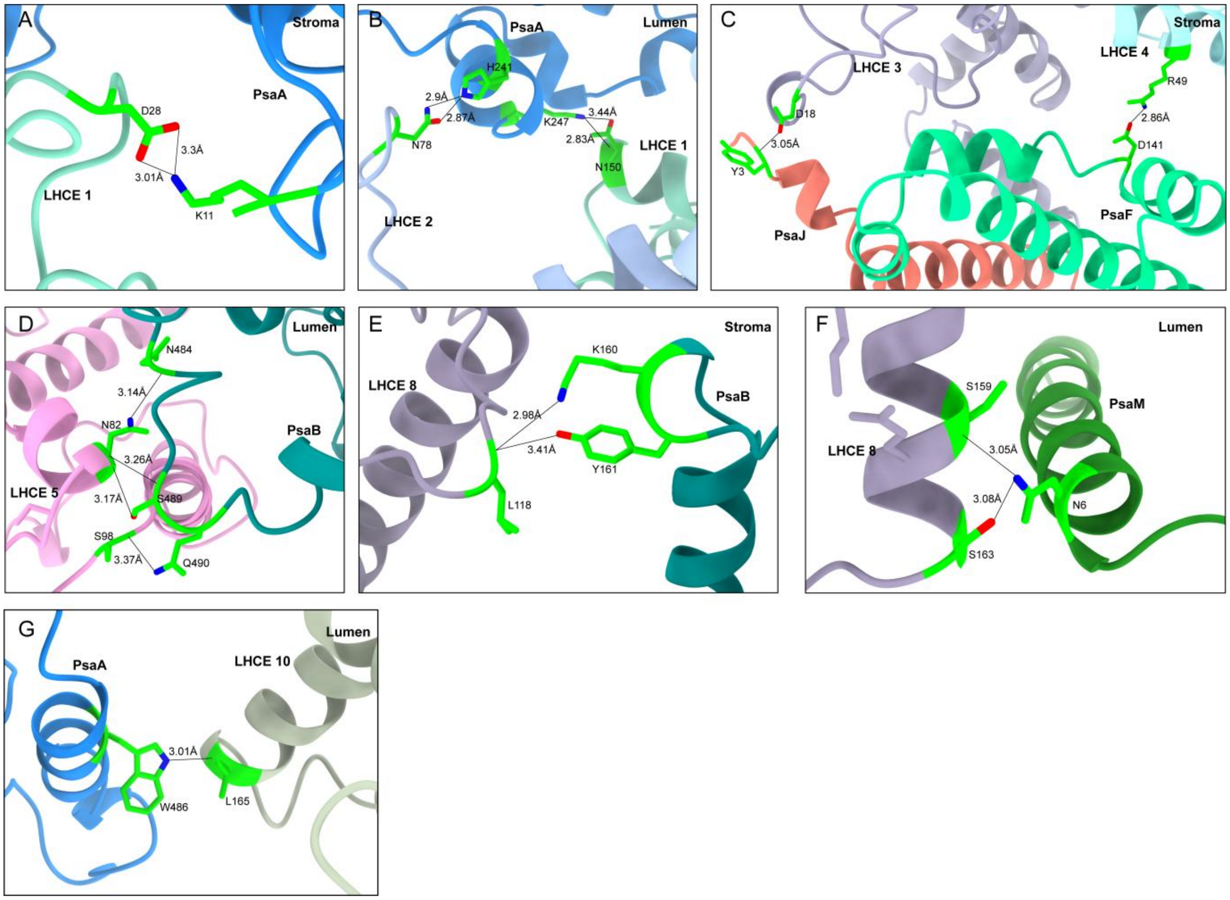

Fig. S16. Interactions between LHCE and core subunits in the PSI-LHCE. (**A**) Interactions between LHCE 1 and PsaA at the stromal side. (**B**) Interactions of PsaA with LHCE 1 and LHCE 2 at the luminal side. (**C**) Interactions of PsaJ with LHCE 3, and PsaF with LHCE 4 at the stromal side. (**D**) Interactions between LHCE 5 and PsaB at the luminal side. (**E**) Interactions between LHCE 8 and PsaB at the stromal side. (**F**) Interactions between LHCE 8 and PsaM at the luminal side. (**G**) Interactions between LHCE 10 and PsaA at the luminal side. Interacting amino acid residues are depicted in green; oxygen (O) and nitrogen (N) atoms are colored red and blue, respectively. All interactions are indicated by solid black lines with distances labeled in Å.

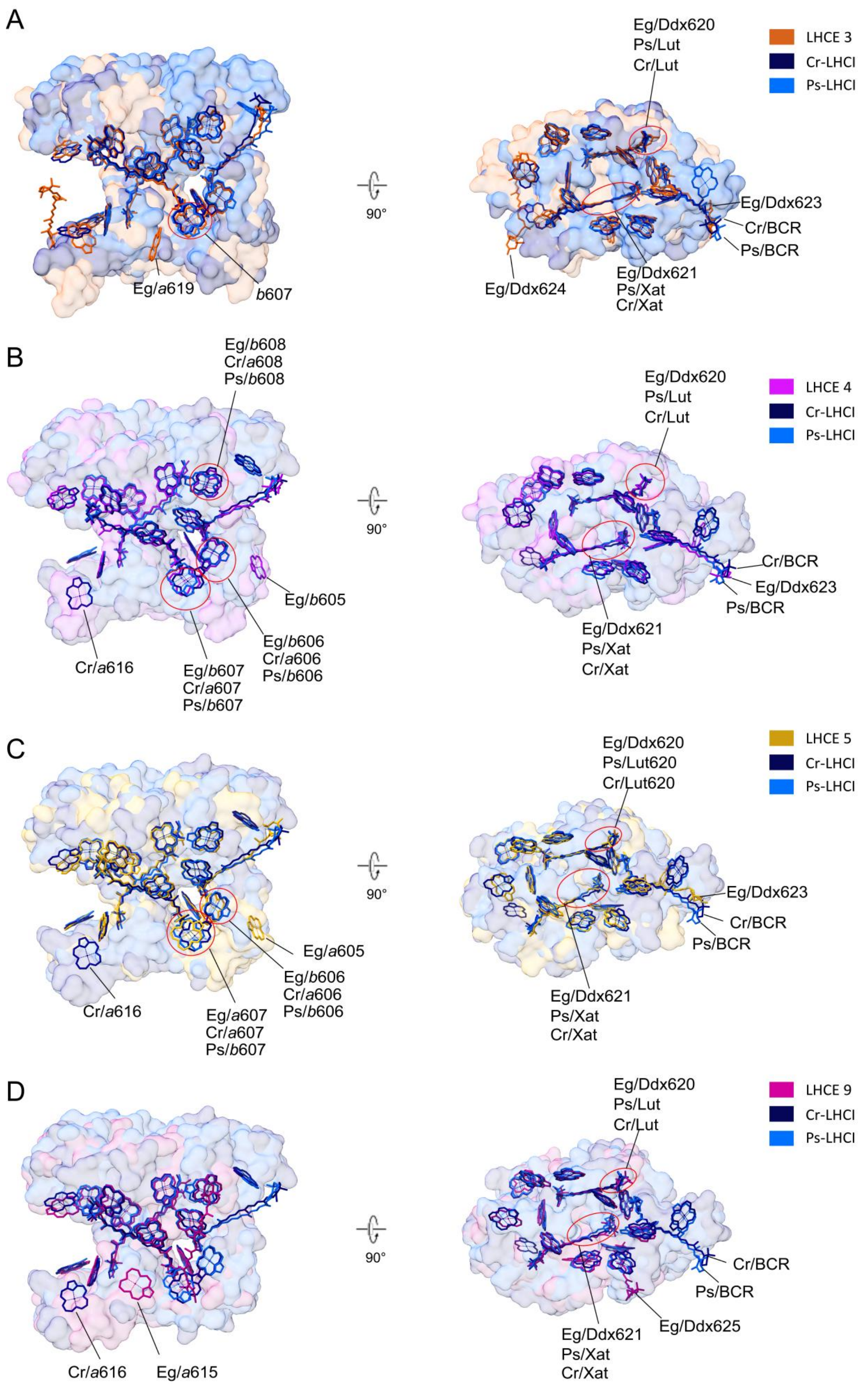

Fig. S17. Comparison of pigment arrangements in *E. gracilis* LHCEs with those in green algal (PDB: 6IJO) and higher plant (PDB: 4XK8) LHCIs. (**A to D**) Superposition of Chl and carotenoid sites in *E. gracilis* LHCEs with those in green algal and plant LHCIs. New pigment sites and absent sites in *E. gracilis* LHCEs are labeled.

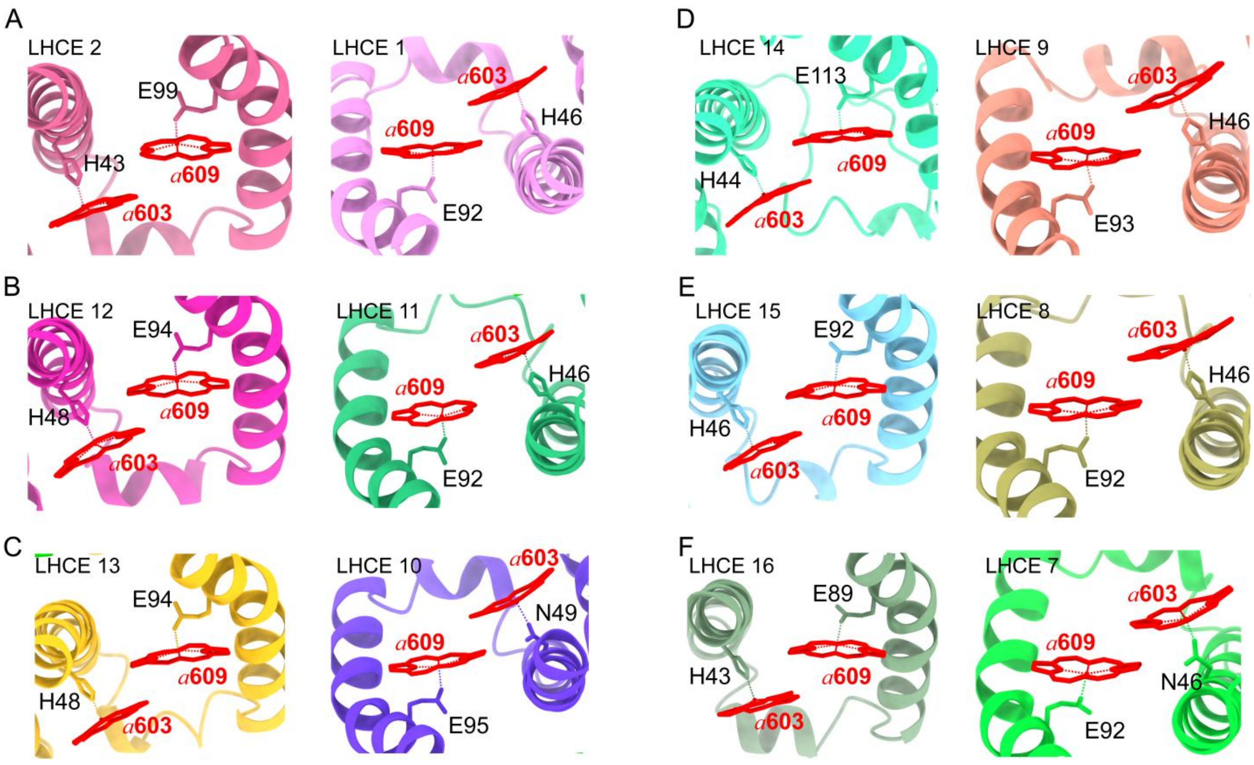

Fig. S18. Coordinating amino acid residues of Chls *a*603–*a*609 in the heterodimers formed by LHCE 1/2 (A), LHCE 11/12 (B), LHCE 10/13 (C), LHCE 9/14 (D), LHCE 8/15 (E), and LHCE 7/14 (F).

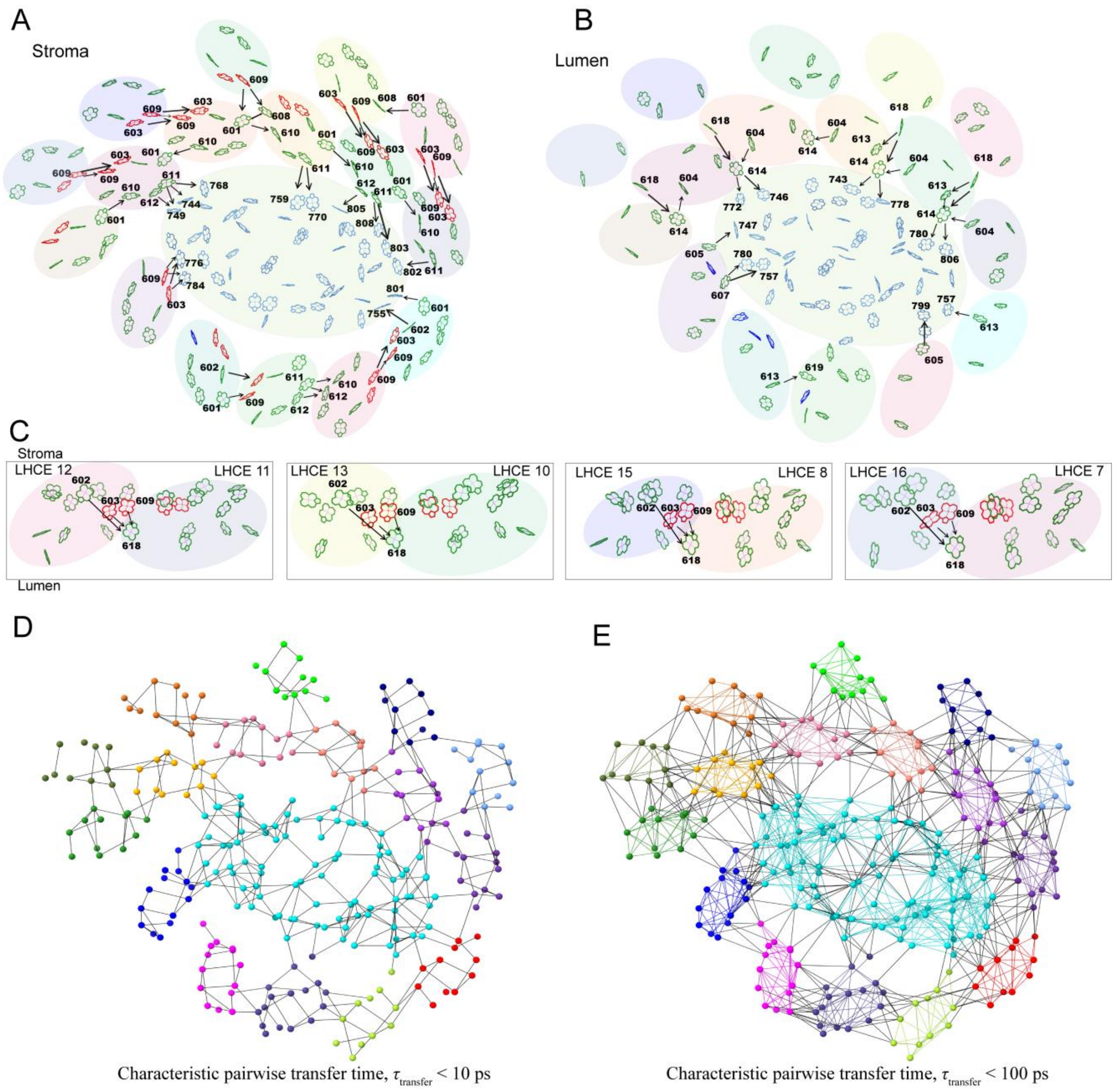

Fig. S19. Pigment arrangement and potential energy transfer pathways in the PSI-LHCE supercomplex. (A) Potential excitation energy transfer (EET) pathways within PSI-LHCE at the stromal side. **(B)** Potential EET pathways within PSI-LHCE at the luminal side. **(C)** Chls involved in energy transfer between the outer and inner antenna. **(D-E)** Organization of Chl molecules and the FRET networks illustrating putative pairwise transfer pathways with characteristic timescales (τ_{transfer}) of less than 10 ps **(D)** and 100 ps **(E)** in the PSI-LHCE supercomplex viewed from the stromal side. Chl a_{LHCE} and Chl b_{LHCE} are shown in green and blue respectively. Chl a_{Core} shown in Cyan. FRET processes between adjacent Chls are shown as lines. Phytyl chains of Chls are omitted for clarity.

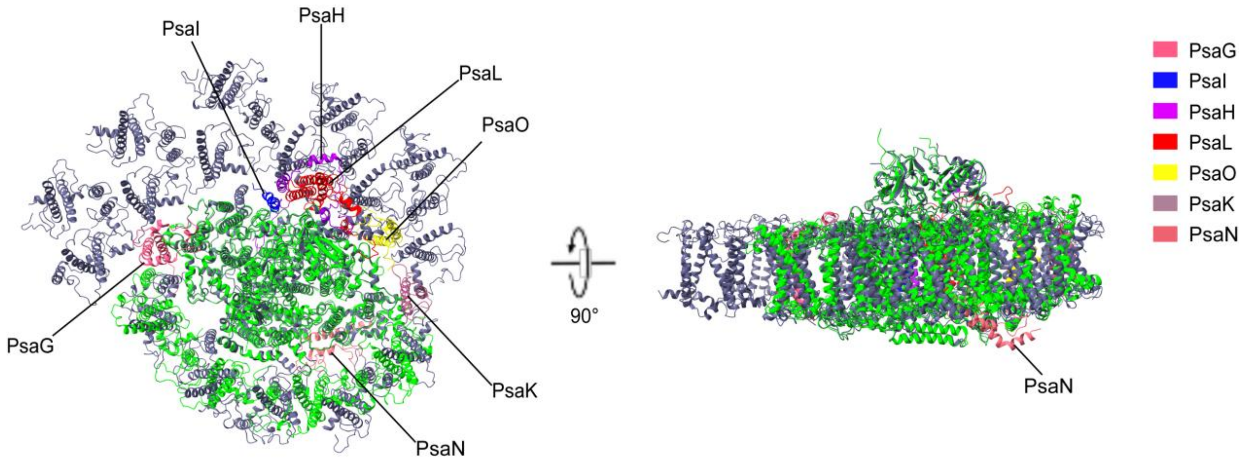

Fig. S20. Superposition of the *E. gracilis* PSI-LHCE with the PSI-LHCI from maize (PDB: 5ZJI). The whole *E. gracilis* PSI-LHCE and the conserved maize PSI core subunits and LHCIs are colored gray and green, respectively. The specific core subunits PsaG, PsaI, PsaH, PsaL, PsaO, PsaK, and PsaN are shown in hot pink, blue, magenta, red, yellow, violet-red, and light coral, respectively.

Table S1. Cryo-EM data collection, refinement, and validation statistics

**Date collection and processing**	**PSI-LHCE (EMD-64823) (PDB 9V7T)**
Magnification	81,000 ×
Voltage(kV)	300
Electron Exposure (e^- /Å^2)	60
Defocus range (μm)	-1.0 ~ -2.0
Pixel size (Å)	1.04
Symmetry imposed	C2
Number of initial particles	5138736
Number of final particles	562173
Map resolution (Å)	2.23
FSC threshold	0.143
B factors (Å^2)	
Protein residues	73.32
Ligands	40.84
Root mean square deviations	
Bond lengths (Å)	0.004
Bond angles (°)	0.637
Validation	
Clashscore	9.43
Rotamer outliers (%)	0.34
Ramachandran plot	
Favored (%)	95.18
Allowed (%)	4.6

Table S2. Protein subunits and cofactors assigned in the structure of *E. gracilis* PSI-LHCE

Subunits	Chain	Ligands
PsaA	A	46Chla,6β-Car, 2Ddx, 2LHG, 1PNQ, 1LMT,1SQD
PsaB	B	41Chla, 6β-Car, 1LMG, 2PNQ, 1DGD, 1SF4
PsaC	C	2SF4
PsaD	D	
PsaE	E	
PsaF	F	1Chla, 1Ddx, 1SQD
PsaJ	J	1Chla, 1Ddx
PsaM	M	1β-Car
LHCE 1: Lhca11	a	11Chla, 2Ddx, 1LMG, 1LHG
LHCE 2: Lhca11	b	11Chla, 2Ddx, 1LHG,1DGD
LHCE 3: Lhcbm3	c	14Chla, 1Chlb, 4Ddx, 1LHG
LHCE 4: Lhcbm1	d	10Chla, 4Chlb, 3Ddx, 1LHG, 1PNQ
LHCE 5: Lhcbm10	e	13Chla, 1Chlb, 3Ddx, 1DGD
LHCE 6: Lhca7	f	12Chla, 2Ddx
LHCE 7: Lhca6	g	13Chla, 2Ddx, 1LHG
LHCE 8: Lhca7	h	13Chla, 2Ddx, 2LHG
LHCE 9: Lhca7	i	12Chla, 3Ddx, 1LMG, 1LHG
LHCE 10: Lhca6	j	13Chla, 2Ddx, 1LHG, 1SQD
LHCE 11: Lhca5	k	13Chla, 2Ddx, 1LMG
LHCE 12: Lhca6	l	12Chla, 2Ddx
LHCE 13: Lhca6	m	10Chla, 2Ddx, 1LHG
LHCE 14: Lhcbm10	n	11Chla, 3Ddx
LHCE 15: Lhca6	o	10Chla, 2Ddx
LHCE 16: Lhca5	p	9Chla, 1Ddx
PSI-LHCE		276Chla, 6Chlb, 13β-Car, 41Ddx, 3SF4, 4PQN<br>1LMT, 4SQD, 4LMG, 12LHG, 3DGD

Table S3. Comparison of protein subunits in the PSI complexes of *E. gracilis*, cyanobacteria, red algae, cryptophytes, diatoms, *Symbiodinium*, green algae, mosses, and higher plants

Subunit	*E. gracilis*	Moss	Green algae	Plants	Cyanobacteria	Red algae	Cryptophyte	*Symbiodinium*	Diatom
PsaA	√	√	√	√	√	√	√	√	√
PsaB	√	√	√	√	√	√	√	√	√
PsaC	√	√	√	√	√	√	√	√	√
PsaD	√	√	√	√	√	√	√	√	√
PsaE	√	√	√	√	√	√	√	√	√
PsaF	√	√	√	√	√	√	√	√	√
PsaG		√	√	√					
PsaH		√	√	√					
PsaI		√	√	√	√	√	√	√	√
PsaJ	√	√	√	√	√	√	√	√	√
PsaK		√	√	√	√	√	√		
PsaL		√	√	√	√	√	√	√	√
PsaM	√	√	√		√	√	√	√	√
PsaN			√	√					
PsaO		√	√	√		√	√		
PsaR						√	√	√	√
PsaS									√
PsaX					√				
PsaT								√	
PsaU								√	
Unk1							√		
ACPI-S							√		
LHCI *n*	16	4 or 8	6，8，9 or 10	4	0	3，5 or 8	11 or 14	13	24

√: present of the subunit.

Table S4. Pigment-binding sites in the LHCE subunits

**LHCE**	**601**	**602**	**603**	**604**	**605**	**606**	**607**	**608**	**609**	**610**	**611**	**612**	**613**	**614**	**615**	**616**	**617**	**618**	**619**	**620**	**621**	**623**	**624**	**625**
LHCE 1	Chl *a*	Chl *a*	Chl *a*	Chl *a*				Chl *a*	Chl *a*	Chl *a*	Chl *a*	Chl *a*	Chl *a*						Chl *a*	Ddx	Ddx			
LHCE 2	Chl *a*	Chl *a*	Chl *a*	Chl *a*	Chl *a*			Chl *a*	Chl *a*	Chl *a*	Chl *a*	Chl *a*	Chl *a*							Ddx	Ddx			
LHCE 3	Chl *a*	Chl *a*	Chl *a*	Chl *a*		Chl *a*	**Chl *b***	Chl *a*	Chl *a*	Chl *a*	Chl a	Chl *a*	Chl *a*	Chl *a*		Chl *a*			Chl *a*	Ddx	Ddx	Ddx	Ddx	
LHCE 4	Chl *a*	Chl *a*	Chl *a*	Chl *a*	**Chl *b***	**Chl *b***	**Chl *b***	**Chl b**	Chl *a*	Chl *a*	Chl *a*	Chl *a*	Chl *a*	Chl *a*						Ddx	Ddx	Ddx		
LHCE 5	Chl *a*	Chl *a*	Chl *a*	Chl *a*	Chl *a*	**Chl *b***	Chl *a*	Chl *a*	Chl *a*	Chl *a*	Chl *a*	Chl *a*	Chl *a*	Chl *a*						Ddx	Ddx	Ddx		
LHCE 6	Chl *a*	Chl *a*	Chl *a*	Chl *a*				Chl *a*	Chl *a*	Chl *a*	Chl *a*	Chl *a*	Chl *a*	Chl *a*	Chl *a*					Ddx	Ddx			
LHCE 7	Chl *a*	Chl *a*	Chl *a*	Chl *a*				Chl *a*	Chl *a*	Chl *a*	Chl *a*	Chl *a*	Chl *a*	Chl *a*	Chl *a*			Chl *a*		Ddx	Ddx			
LHCE 8	Chl *a*	Chl *a*	Chl *a*	Chl *a*				Chl *a*	Chl *a*	Chl *a*	Chl *a*	Chl *a*	Chl *a*	Chl *a*	Chl *a*			Chl *a*		Ddx	Ddx			
LHCE 9	Chl *a*	Chl *a*	Chl *a*	Chl *a*				Chl *a*	Chl *a*	Chl *a*	Chl *a*	Chl *a*	Chl *a*	Chl *a*	Chl *a*					Ddx	Ddx			Ddx
LHCE 10	Chl *a*	Chl *a*	Chl *a*	Chl *a*				Chl *a*	Chl *a*	Chl *a*	Chl *a*	Chl *a*	Chl *a*	Chl *a*	Chl *a*			Chl *a*		Ddx	Ddx			
LHCE 11	Chl *a*	Chl *a*	Chl *a*	Chl *a*				Chl *a*	Chl *a*	Chl *a*	Chl *a*	Chl *a*	Chl *a*	Chl *a*	Chl *a*			Chl *a*		Ddx	Ddx			
LHCE 12	Chl *a*	Chl *a*	Chl *a*	Chl *a*				Chl *a*	Chl *a*	Chl *a*	Chl *a*	Chl *a*	Chl *a*	Chl *a*			Chl *a*			Ddx	Ddx			
LHCE 13	Chl *a*	Chl *a*	Chl *a*	Chl *a*				Chl *a*	Chl *a*	Chl *a*	Chl *a*	Chl *a*	Chl *a*							Ddx	Ddx			
LHCE 14		Chl *a*	Chl *a*	Chl *a*	Chl *a*	Chl *a*	Chl *a*	Chl *a*	Chl *a*	Chl *a*		Chl *a*	Chl *a*							Ddx	Ddx	Ddx		
LHCE 15	Chl *a*	Chl *a*	Chl *a*	Chl *a*				Chl *a*	Chl *a*	Chl *a*		Chl *a*	Chl *a*	Chl *a*						Ddx	Ddx			
LHCE 16	Chl *a*	Chl *a*	Chl *a*	Chl *a*				Chl *a*	Chl *a*	Chl *a*		Chl *a*	Chl *a*								Ddx			

Table S5. Possible EET pathways between LHCEs based on the Förster theory

Code	Pathway	Pigments	FRET rate constants, k_{FRET} (ps^{-1})	Characteristic pairwise transfer time, τ_{transfer} (ps)	Distance, R(Å)	Dipole orientation factor, κ^2
1-2$_{stroma}$	LHCE 1 - LHCE 2	Chl a609$_{LHCE\ 1}$ - a609$_{LHCE\ 2}$<br>Chl a609$_{LHCE\ 1}$ - a603$_{LHCE\ 2}$<br>Chl a603$_{LHCE\ 1}$ - a609$_{LHCE\ 2}$	2.463<br>0.168<br>0.161	0.406<br>5.949<br>6.196	14.305<br>22.415<br>22.321	3.776<br>3.814<br>3.571
2-3$_{stroma}$	LHCE 2 - LHCE 3	Chl a612$_{LHCE\ 2}$ - a611$_{LHCE\ 3}$<br>Chl a612$_{LHCE\ 2}$ - a612$_{LHCE\ 3}$<br>Chl a610$_{LHCE\ 2}$ - a611$_{LHCE\ 3}$	0.200<br>0.386<br>0.393	4.988<br>2.592<br>2.545	12.612<br>14.353<br>15.635	0.144<br>0.603<br>1.027
3-4$_{stroma}$<br>**3-4$_{Lumen}$**	LHCE 3 - LHCE 4	Chl a609$_{LHCE\ 3}$ - a601$_{LHCE\ 4}$<br>Chl a609$_{LHCE\ 3}$ - a602$_{LHCE\ 4}$<br>**Chl a619$_{LHCE\ 3}$ - a613$_{LHCE\ 4}$**	2.451<br>0.131<br>**0.342**	0.408<br>7.632<br>**2.923**	11.900<br>17.214<br>**17.986**	1.245<br>0.610<br>**2.073**
6-7$_{stroma}$<br>**6-7$_{Lumen}$**	LHCE 6 - LHCE 7	Chl a601$_{LHCE\ 6}$ - a610$_{LHCE\ 7}$<br>**Chl a614$_{LHCE\ 6}$ - a604$_{LHCE\ 7}$**<br>**Chl a614$_{LHCE\ 6}$ - a608$_{LHCE\ 7}$**<br>**Chl a614$_{LHCE\ 6}$ - a618$_{LHCE\ 7}$**	0.520<br>**0.165**<br>**0.102**<br>**0.056**	1.922<br>**6.057**<br>**9.813**<br>**17.845**	17.707<br>**15.132**<br>**20.963**<br>**23.113**	2.87<br>**0.355**<br>**1.547**<br>**1.529**
7-8$_{stroma}$<br>**7-8$_{Lumen}$**	LHCE 7 - LHCE 8	Chl a601$_{LHCE\ 7}$ - a610$_{LHCE\ 8}$<br>**Chl a614$_{LHCE\ 7}$ - a604$_{LHCE\ 8}$**<br>**Chl a613$_{LHCE\ 7}$ - a608$_{LHCE\ 8}$**	0.599<br>**1.436**<br>**0.638**	1.670<br>**0.696**<br>**1.567**	17.399<br>**13.214**<br>**15.957**	2.973<br>**1.367**<br>**1.884**
8-9$_{stroma}$<br>**8-9$_{Lumen}$**	LHCE 8 - LHCE 9	Chl a601$_{LHCE\ 8}$ - a608$_{LHCE\ 9}$<br>Chl a601$_{LHCE\ 8}$ - a610$_{LHCE\ 9}$<br>**Chl a614$_{LHCE\ 8}$ - a604$_{LHCE\ 9}$**<br>**Chl a614$_{LHCE\ 8}$ - a608$_{LHCE\ 9}$**	2.531<br>0.465<br>**1.436**<br>**0.102**	0.395<br>2.148<br>**0.697**<br>**9.812**	12.213<br>17.360<br>**13.214**<br>**20.537**	1.503<br>2.280<br>**1.367**<br>**1.368**
9-10$_{stroma}$<br>**9-10$_{Lumen}$**	LHCE 9 - LHCE 10	Chl a601$_{LHCE\ 9}$ - a610$_{LHCE\ 10}$<br>**Chl a614$_{LHCE\ 9}$ - a604$_{LHCE\ 10}$**<br>**Chl a614$_{LHCE\ 9}$ - a608$_{LHCE\ 10}$**<br>**Chl a613$_{LHCE\ 9}$ - a618$_{LHCE\ 10}$**	0.624<br>**0.159**<br>**0.106**<br>**0.670**	1.601<br>**6.261**<br>**9.410**<br>**1.493**	17.480<br>**15.307**<br>**21.123**<br>**16.033**	3.188<br>**0.367**<br>**1.689**<br>**2.036**
10-11$_{stroma}$<br>**10-11$_{Lumen}$**	LHCE 10 - LHCE 11	Chl a601$_{LHCE\ 10}$ - a610$_{LHCE\ 11}$<br>**Chl a614$_{LHCE\ 10}$ - a604$_{LHCE\ 11}$**<br>**Chl a613$_{LHCE\ 10}$ - a618$_{LHCE\ 11}$**	0.615<br>**0.158**<br>**0.611**	1.626<br>**6.332**<br>**1.637**	17.602<br>**15.209**<br>**15.828**	3.272<br>**0.350**<br>**1.718**
12-11$_{stroma}$<br>***12-11$_{stroma\text{-}Lumen}$***	LHCE 12 - LHCE 11	Chl a609$_{LHCE\ 12}$ - a609$_{LHCE\ 11}$<br>Chl a609$_{LHCE\ 12}$ - a603$_{LHCE\ 11}$<br>Chl a603$_{LHCE\ 12}$ - a609$_{LHCE\ 11}$<br>***Chl a609$_{LHCE\ 12}$/Chl a603$_{LHCE\ 12}$/Chl a602$_{LHCE\ 12}$ - Chl a618$_{LHCE\ 11}$***	2.302<br>0.147<br>0.162<br>***0.630/1.228/0.122***	0.434<br>6.780<br>6.157<br>***1.586/0.814/8.203***	14.484<br>22.759<br>22.367<br>***11.606/11.646/23.109***	3.802<br>3.668<br>3.638<br>***0.276/0.548/3.322***

12-13$_{stroma}$	LHCE 12 - LHCE 13	Chl a601$_{LHCE\ 12}$ - a608$_{LHCE\ 13}$	0.223	4.489	19.745	2.362
13-10$_{stroma}$<br>***13-10$_{stroma\text{-}Lumen}$***	LHCE 13 - LHCE 10	Chl a609$_{LHCE\ 13}$ - a609$_{LHCE\ 10}$<br>Chl a609$_{LHCE\ 13}$ - a603$_{LHCE\ 10}$<br>Chl a603$_{LHCE\ 13}$ - a609$_{LHCE\ 10}$<br>***Chl a609$_{LHCE\ 13}$/Chl a603$_{LHCE\ 13}$/Chl a602$_{LHCE\ 13}$ - a618$_{LHCE\ 10}$***	2.408<br>0.155<br>0.171<br>***0.527/1.177/0.117***	0.415<br>6.437<br>5.841<br>***1.899/0.849/8.575***	14.435<br>22.681<br>22.234<br>***11.798/11.605/23.069***	3.898<br>3.785<br>3.701<br>***0.254/0.515/3.145***
14-9/8$_{stroma}$	LHCE 14 - LHCE 9/LHCE 8	Chl a609$_{LHCE\ 14}$ - a608$_{LHCE\ 9}$/a601$_{LHCE\ 8}$	0.289/0.148	3.454/6.739	19.239/19.577	2.627/1.495
15-8$_{stroma}$<br>***15-8$_{stroma\text{-}Lumen}$***	LHCE 15 - LHCE 8	Chl a609$_{LHCE\ 15}$ - a609$_{LHCE\ 8}$<br>Chl a609$_{LHCE\ 15}$ - a603$_{LHCE\ 8}$<br>Chl a603$_{LHCE\ 15}$ - a609$_{LHCE\ 8}$<br>***Chl a609$_{LHCE\ 15}$/Chl a603$_{LHCE\ 15}$/Chl a602$_{LHCE\ 15}$ - a618$_{LHCE\ 8}$***	2.216<br>0.152<br>0.167<br>***0.668/1.206/0.115***	0.451<br>6.597<br>5.989<br>***1.496/0.829/8.700***	14.633<br>22.762<br>22.361<br>***11.808/11.385/23.040***	3.893<br>3.772<br>3.735<br>***0.324/0.470/3.077***
16-7$_{stroma}$<br>***16-7$_{stroma\text{-}Lumen}$***	LHCE 16 - LHCE 7	Chl a609$_{LHCE\ 16}$ - a609$_{LHCE\ 7}$<br>Chl a609$_{LHCE\ 16}$ - a603$_{LHCE\ 7}$<br>***Chl a609$_{LHCE\ 16}$/Chl a602$_{LHCE\ 16}$ - a618$_{LHCE\ 7}$***	2.387<br>0.145<br>***0.342/0.122***	0.419<br>6.883<br>***2.920/8.183***	14.442<br>22.769<br>***11.753/23.161***	3.876<br>3.622<br>***0.162/3.375***

Possible EET pathways between LHCEs at the lumenal side are indicated in bold text, and the special EET pathways from the stromal side to the luminal side within four LHCE heterodimer (LHCE 12/11, LHCE 13/10, LHCE 15/8, LHCE 16/7) are indicated in bold italic text.

Table S6. Possible EET pathways form LHCEs to the PSI core based on the Förster theory

Code	Pathway	Pigments	FRET rate constants, k_{FRET} (ps^{-1})	Characteristic pairwise transfer time, $\tau_{transfer}$ (ps)	Distance, R(Å)	Dipole orientation factor, κ^2
1-A$_{stroma}$<br>**1-A$_{Lumen}$**	LHCE 1 - PsaA	Chl a602$_{LHCE\ 1}$ - a755$_{PsaA}$<br>Chl a601$_{LHCE\ 1}$ - a801$_{PsaA}$<br>**Chl a613$_{LHCE\ 1}$ - a757$_{PsaA}$**	0.065<br>0.216<br>**0.439**	15.296<br>4.634<br>**2.279**	16.558<br>14.453<br>**15.99**	0.241<br>0.352<br>**1.312**
**2-A$_{Lumen}$**	**LHCE 2 - PsaA**	**Chl a605$_{LHCE\ 2}$ - a799$_{PsaA}$**	**0.436**	**2.291**	**17.178**	**2.006**
**3-F$_{Lumen}$**	**LHCE 3 - PsaA**	**Chl a616$_{LHCE\ 3}$ - a793$_{PsaA}$**	**0.114**	**8.736**	**19.843**	**1.250**
5-B$_{stroma}$<br>**5-B$_{Lumen}$**	LHCE 5 - PsaB	Chl a603/609$_{LHCE\ 5}$ - a784$_{PsaB}$<br>Chl a603/609$_{LHCE\ 5}$ - a776$_{PsaB}$<br>**Chl a607$_{LHCE\ 5}$ - a757/780$_{PsaB}$**<br>**Chl a605$_{LHCE\ 5}$ - a747$_{PsaB}$**	0.444/0.361<br>0.155/0.391<br>**0.199/1.974**<br>**0.297**	2.250/2.773<br>6.463/2.558<br>**5.006/0.506**<br>**3.368**	15.074/12.224<br>20.789/14.012<br>**20.137/13.485**<br>**15.536**	0.933/0.215<br>2.235/0.529<br>**2.383/2.124**<br>**0.747**
7-B$_{stroma}$<br>**7-B$_{Lumen}$**	LHCE 7 - PsaB	Chl a611$_{LHCE\ 7}$ - a768/a744/a749$_{PsaB}$<br>Chl a612$_{LHCE\ 7}$ - a749$_{PsaB}$<br>**Chl a614$_{LHCE\ 7}$ - a746/772$_{PsaB}$**	0.146/0.140/0.271<br>0.121<br>**0.121/0.134**	6.853/7.136/3.688<br>8.266<br>**8.292/7.481**	18.348/12.574/15.162<br>12.936<br>**19.030/16.404**	0.996/0.099/0.589<br>0.101<br>**1.025/0.466**
9-B$_{stroma}$<br>**9-A/B$_{Lumen}$**	LHCE 9 - PsaB/PsaA	Chl a611$_{LHCE\ 9}$ - a759/770$_{PsaB}$<br>**Chl a614$_{LHCE\ 9}$ - a778$_{PsaA}$**<br>**Chl a614$_{LHCE\ 9}$ - a743$_{PsaB}$**	0.102/0.173<br>**0.499**<br>**0.605**	9.811/5.776<br>**2.003**<br>**1.653**	21.595/21.098<br>**16.828**<br>**16.131**	1.849/2.732<br>**2.029**<br>**1.907**
10-B$_{stroma}$<br>**10-B$_{Lumen}$**	LHCE 10 - PsaA	Chl a612$_{LHCE\ 10}$ - a805$_{PsaA}$<br>Chl a611$_{LHCE\ 10}$ - a803/808$_{PsaA}$<br>**Chl a614$_{LHCE\ 10}$ - a780/806$_{PsaA}$**	0.389<br>0.110/0.109<br>**0.310/0.272**	2.568<br>9.078/9.134<br>**3.224/3.681**	15.731<br>14.841/23.679<br>**16.451/17.132**	1.056<br>0.209/3.474<br>**1.100/1.229**
11-A$_{stroma}$	LHCE 11 - PsaA	Chl a611$_{LHCE\ 11}$ - a802$_{PsaA}$	0.148	6.773	18.028	0.907
